# An Efficient Workflow for Fungal Nucleic Acid Detection in Sputum

**DOI:** 10.3390/jof12040252

**Published:** 2026-03-31

**Authors:** Ying Han, Deding Tang, Yuntong Gao, Lilian Ji, Jie Hu, Yan Gong

**Affiliations:** 1 School of Chemistry and Life Sciences, Suzhou University of Science and Technology, Suzhou 215009, China; hying8173@163.com (Y.H.); jll2663@sina.com (L.J.); 2 The Key Laboratory of Biomedical Information Engineering of Ministry of Education, School of Life Science and Technology, Xi’an Jiaotong University, Xi’an 710049, China; 10085@massz.edu.cn; 3 Bioinspired Engineering and Biomechanics Center (BEBC), Xi’an Jiaotong University, Xi’an 710049, China; 4 Public Teaching Department, Maanshan Teacher’s College, Maanshan 243041, China; 5 Suzhou Diyinan Biotech Company, Suzhou 215129, China; gaoyt7777@163.com

**Keywords:** respiratory fungal infections, pathogenic fungi cell wall, nucleic acid extraction, multiplex real-time qPCR, early diagnosis

## Abstract

Respiratory fungal infections are challenging to diagnose due to non-specific symptoms and limitations of conventional methods. This study established an integrated, rapid workflow for detecting three common pathogenic fungi, combining an optimized nucleic acid extraction method with a TaqMan-based multiplex real-time qPCR system. The high-salt lysis method yielded high-purity nucleic acid extracts, with performance comparable to or better than commercial kits. The multiplex qPCR assay was optimized for high specificity, sensitivity, and reproducibility, with detection limits as low as 10 CFU/mL for some fungi. Clinical validation using 14 sputum samples demonstrated high agreement with conventional culture, and the assay detected a low-load infection missed by culture. This integrated approach shows great potential for rapid, sensitive, and specific clinical detection of respiratory fungal infections.

## 1. Introduction

Fungi, as ubiquitously distributed eukaryotic organisms, pose a substantial threat to human health via their pathogenic strains. To date, hundreds of fungal species have been identified as etiological agents of human infectious diseases [1], among which invasive fungal infections (IFIs) constitute a pressing global public health concern owing to their rapid progression and unfavorable prognosis. It is estimated that over 150 million individuals worldwide are afflicted with IFIs [2,3], resulting in more than 1.6 million deaths annually [4]. Notably, the incidence of IFIs continues to escalate, with immunocompromised populations facing a significantly elevated risk. In the context of pulmonary IFIs, *Candida albicans (C. albicans)*, *Aspergillus fumigatus (A. fumigatus)*, and *Cryptococcus neoformans* *(C. neoformans)* have been categorized by the World Health Organization (WHO) as “critical-priority” pathogens [5]. Specifically, their infection rates in intensive care units (ICUs) stand at 43.2%, 15.2–24.2%, and 0.2–0.9 per 100,000 individuals, respectively. For untreated severe cryptococcosis, the associated mortality rate can be as high as 100% [6,7], highlighting the imperative for timely clinical intervention.

Early and precise etiological diagnosis is crucial for reducing IFI-related mortality [8]. However, current clinical practice still relies heavily on traditional microbial culture methods [9], which suffer from two major limitations: poor timeliness (3–5 days for standard culture, with longer durations required for slow-growing strains [10]) and low sensitivity (detection rates of only 20% in patients pretreated with antifungal drugs and less than 30% in cases of mixed infections [11,12]). These shortcomings lead to significant delays in initiating appropriate treatment. Serological tests, such as the (1 → 3)-β-D-glucan test (G test) and galactomannan test (GM test) [13], offer rapid results but have limitations: their specificity can be easily compromised, and they cannot replace direct pathogen confirmation.

Molecular diagnostic techniques, including quantitative polymerase chain reaction (qPCR), recombinase polymerase amplification (RPA), and loop-mediated isothermal amplification (LAMP) [14], hold great potential for overcoming existing diagnostic bottlenecks. Among these, multiplex PCR represents a particularly valuable advancement, enabling the simultaneous detection and differentiation of multiple target pathogens in a single reaction. This approach not only improves diagnostic efficiency and reduces turnaround time but also enhances the detection of mixed infections, which are common in pulmonary IFIs. By incorporating species-specific probes or primers, multiplex qPCR achieves high specificity, making it a promising tool for clinical application in resource-limited settings [15]. However, a major challenge in the clinical translation of these molecular methods lies in the extraction of fungal nucleic acids from sputum samples.

Sputum was selected as the primary clinical sample in this study for several reasons. First, it is collected non-invasively and is readily accessible, making it suitable for routine clinical screening. Second, sputum directly reflects the microbial composition of the lower respiratory tract, providing a direct window into the etiology of pulmonary infections [16]. However, sputum also presents notable disadvantages: it is highly viscous due to the presence of mucins, which can impede nucleic acid extraction; it contains host cells, debris, and potential contaminants that may interfere with amplification reactions; and it may not always be the optimal sample for certain fungal pathogens, as some may colonize the upper respiratory tract without causing active disease [17]. Despite these limitations, sputum remains the most practical and widely used sample for the diagnosis of respiratory infections in clinical practice.

Sputum contains components such as mucins, host cells, and drug degradation products that can inhibit nucleic acid amplification; additionally, the fungal cell wall—composed of chitin and β-glucan—is difficult to lyse effectively [18]. Existing commercial kits fail to address these challenges [16], resulting in low nucleic acid extraction efficiency and poor purity, both of which negatively impact detection accuracy.

Fungal cell wall architecture is the core factor restricting the efficient lysis of fungal cells in sputum samples, and the three WHO critical-priority pathogenic fungi selected in this study have distinct cell wall structural characteristics: *C. albicans* has a thick multi-layered cell wall composed of chitin and β-glucan, *C. neoformans* has a unique polysaccharide capsule with moderate permeability, and *A. fumigatus* has a thin and porous filamentous fungal cell wall. Based on these structural characteristics, we proposed a fungal cell wall architecture-driven optimization hypothesis: a tailored lysis system combining high-concentration denaturant, anionic surfactant and alkaline environment can specifically disrupt the cell wall/capsule structure of different fungi, balance the efficiency of cell lysis and the stability of nucleic acid, and thus improve the extraction efficiency and purity of fungal nucleic acid from sputum samples. Based on this hypothesis, we designed and optimized the key components of the lysis buffer and further constructed a multiplex qPCR detection system for the simultaneous detection of the three fungi.

In summary, establishing an efficient system for nucleic acid extraction and rapid detection of fungal pathogens in sputum samples is of immense value for the early diagnosis of pulmonary IFIs, guiding clinical therapy, and reducing associated mortality. This study addresses two key technological bottlenecks through innovative approaches: first, the development of a customized fungal nucleic acid extraction protocol to overcome the limitations of current methodologies; second, the construction of a single-tube triple qPCR assay based on this optimized protocol, which enables the simultaneous detection of *C. albican*, *A. fumigatus*, and *C. neoformans*. Together, these innovations provide a reliable molecular diagnostic solution for clinical application.

## 2. Experimental Section

### 2.1. Chemicals and Materials

The reagents employed in this experiment were as follows: Tween-20, Triton X-100, dithiothreitol (DTT), guanidine hydrochloride, isopropanol (Shanghai Macklin Biochemical Co., Ltd., Shanghai, China); ethylenediaminetetraacetic acid disodium salt (EDTA-Na_2_), NaOH, chitinase, glucanase, NaCl, glycine (Sinopharm Chemical Reagent Co., Ltd., Shanghai, China); Trizma sodium acetate, sodium octanoate (Sigma-Aldrich (Shanghai) Trading Co., Ltd., Shanghai, China); TAE buffer, 6× glycerol gel loading buffer, DNA molecular weight marker (Sangon Biotech (Shanghai) Co., Ltd., Shanghai, China); 4S GelRed (BBI Life Sciences Ltd., Shanghai, China); agarose (Shanghai Tianneng Technology Co., Ltd., Shanghai, China); high-purity pectin (Xinjiang Fufeng Biotechnology Co., Ltd., Urumqi, China); ordinary sodium carboxymethyl cellulose (CMC-Na, Shandong Fengtai Biotechnology Co., Ltd., Jinan, China); fungal nucleic acid extraction and purification kit (Hangzhou Dahua Blue Biotechnology Co., Ltd., Hangzhou, China). Strains: *C. neoformans* (B82120), *C. albicans* (B84199), *A. fumigatus* (BMZ315652) (all from Ningbo Mingzhou Biotechnology Co., Ltd., Ningbo, China).

The main laboratory instruments utilized included: a biological safety cabinet (model HFaafe-1200LC (B2), Heal Force Bio-Meditech Holdings Limited, Shanghai, China); a high-speed refrigerated centrifuge (model Legend Micro 21R, Thermo Fisher Scientific, Shanghai, China); an automated nucleic acid extractor (model Auto-Pure Mini, Hangzhou Aosense Instrument Co., Ltd., Hangzhou, China); a microspectrophotometer (model Nano-500, Hangzhou Aosense Instrument Co., Ltd., Hangzhou, China); an automated medical PCR analysis system (model SLAN-96P, Shanghai Hongshi Medical Technology Co., Ltd., Shanghai, China); a gel imaging system (model Tannon-1600B, Shanghai Tianneng Technology Co., Ltd., Shanghai, China); and a constant-temperature water bath (model HHS-4, Beijing Time Instrument Technology Co., Ltd., Beijing, China).

### 2.2. Preparation of Simulated Sputum

Artificial sputum was prepared with modifications based on previously reported protocols [17]. The formulation consisted of 1.5% pectin, 0.5% sodium carboxymethyl cellulose (CMC-Na), 0.9% sodium chloride (NaCl), and sterile deionized water. Briefly, the components were mixed in the specified proportions, incubated in an 80 °C water bath with continuous stirring until fully dissolved and homogenized, forming a gel-like substance that mimics the appearance and physical properties (especially viscosity) of clinical sputum. The prepared simulated sputum was immediately transferred to a 4 °C refrigerator for sealed storage, with the storage period strictly adhering to experimental quality control requirements.

### 2.3. Study Subjects

The study subjects were selected from patients with suspected pulmonary invasive fungal infections (IFIs) admitted to the Department of Respiratory Medicine and Intensive Care Unit (ICU) of the Second Affiliated Hospital of Xi’an Jiaotong University from June 2025 to October 2025. All subjects were enrolled in accordance with strict inclusion and exclusion criteria, and the study protocol was approved by the Ethics Committee of the Second Affiliated Hospital of Xi’an Jiaotong University. All patients or their legally authorized representatives signed written informed consent prior to sample collection, ensuring the compliance and ethicality of the study.

#### 2.3.1. Inclusion Criteria

Patients who met the clinical diagnostic criteria for suspected pulmonary IFIs, including but not limited to: having clinical symptoms such as persistent cough, expectoration, fever (body temperature ≥ 38.5°C for more than 3 days), chest pain, and dyspnea; and having positive clues in imaging examinations (chest X-ray or CT showing pulmonary infiltration, nodules, or cavity lesions) that suggested fungal infection. Patients aged 18–75 years old, regardless of gender, with complete clinical data (including medical history, physical examination results, laboratory test reports, and imaging data). Patients who had not received antifungal drug treatment within 72 h before sample collection (to avoid the impact of drug use on fungal detection results). Patients who were able to provide qualified sputum samples (sputum volume ≥ 1 mL, with no obvious contamination by saliva or nasal discharge).

#### 2.3.2. Exclusion Criteria

Patients with other severe diseases that may affect the study results, including severe liver and kidney dysfunction, severe cardiovascular and cerebrovascular diseases, malignant tumors in the advanced stage, and autoimmune diseases. Patients who had received antifungal drug treatment within 72 h before sample collection, or had taken immunosuppressants (such as glucocorticoids, cyclophosphamide) for a long time. Patients with bacterial pneumonia, viral pneumonia, or other non-fungal respiratory infections confirmed by etiological examination. Patients with mental disorders, poor compliance, or inability to cooperate with sample collection and follow-up. Pregnant or lactating women, and patients with allergies to the reagents used in the study.

A total of 14 patients who met the above criteria were finally enrolled in the study, and sputum samples were collected for subsequent experimental verification.

### 2.4. Establishment and Optimization of an Efficient Fungal Nucleic Acid Extraction Method

An optimized high-salt extraction method was developed based on a pre-established protocol, coupled with single-factor optimization experiments. The core workflow included three key steps: (1) pretreatment of simulated sputum via physical ultrasound to partially disrupt fungal cell walls; (2) complete fungal cell lysis and protein denaturation using a combination of high-efficiency guanidine salt denaturant and surfactants; (3) removal of residual impurities through washing and purification to obtain high-purity nucleic acids. To identify critical factors influencing extraction efficiency, a single-variable control design was adopted, wherein only one target factor was adjusted at a time while other conditions remained consistent with the standard operating procedure (SOP). All experiments were performed in triplicate, and the extracted nucleic acids were eluted with an equal volume of nuclease-free water.

#### 2.4.1. Optimization Factors and Gradient Settings

To systematically optimize the nucleic acid extraction efficiency, single-factor variable design was employed. The key optimization parameters, gradient settings, and experimental design are summarized in Table 1.

Guanidine hydrochloride concentration: five gradients (1, 2, 3, 4, and 5 mol/L) were set to evaluate its effects on fungal cell lysis and protein denaturation. This parameter was selected because guanidinium salt serves as the core denaturant in the high-salt method, directly influencing lysis efficiency by disrupting fungal cell membranes and protein structures. Appropriate concentration is critical for balancing sufficient cell lysis and minimizing nucleic acid degradation.

Isopropanol volume fraction: five gradients (30%, 40%, 50%, 60%, and 70%) were used to investigate its influence on nucleic acid precipitation and recovery yield. This factor was chosen as it is the primary precipitant for nucleic acids; its volume fraction directly determines the efficiency of DNA precipitation and the co-precipitation of impurities such as salts and proteins, thereby affecting final yield and purity.

Triton X-100 volume fraction: six gradients (0% as blank control, 2%, 4%, 6%, 8%, and 10%) were applied to assess its role in disrupting fungal cell walls and membranes. This non-ionic detergent was optimized because it aids in dissolving lipids and loosening the fungal cell wall structure. Adjusting its concentration allows for the optimization of cell lysis efficiency without compromising the integrity of the extracted DNA.

System pH: seven gradients (6, 7, 8, 9, 10, 11, and 12) were set to explore the effects of pH on nucleic acid stability and extraction efficiency. pH was identified as a key variable since it directly affects the solubility of nucleic acids, the activity of co-extracted nucleases, and the charge interaction between nucleic acids and precipitants. Optimizing the pH ensures a stable environment that preserves DNA integrity while maximizing extraction efficiency.

All optimization experiments were performed in triplicate, with only one variable changed at a time while other parameters remained constant.

#### 2.4.2. Evaluation Criteria

Nucleic acid purity and concentration were quantified using a NanoDrop microspectrophotometer. Purity was determined by the OD_260_/_280_ (acceptable range: 1.8–2.0), while extraction efficiency was reflected by the measured nucleic acid concentration. For each factor, the gradient yielding the highest nucleic acid concentration (among purity-qualified samples) was defined as the optimal level. The optimized high-salt extraction formula and standardized operating procedure were finalized by integrating the optimal levels of all factors.

#### 2.4.3. Comparison with Commercial Nucleic Acid Extraction Kits

The optimized high-salt method was compared with a commercial fungal DNA extraction kit (magnetic bead-based, Hangzhou Dahua Blue Biotechnology Co., Ltd., Hangzhou, China), which has been reported to have comparable nucleic acid extraction performance with international mainstream kits (e.g., Qiagen, Thermo Fisher, Shanghai, China) in previous studies [18,19,20]. Nucleic acid extraction using the commercial kit was performed according to the manufacturer’s SOP, leveraging magnetic bead-specific binding for purification. Simulated sputum samples were spiked with three concentrations of target fungi: *C. albicans* and *C. neoformans* at low (10^3^ CFU/mL), medium (10^5^ CFU/mL), and high (10^7^ CFU/mL) levels; *A. fumigatus* at low (10^2^ CFU/mL), medium (10^4^ CFU/mL), and high (10^6^ CFU/mL) levels. Nucleic acids were extracted from these spiked samples using both the high-salt method and the commercial kit, with three parallel replicates per method. All extracts were eluted with equal volumes of nuclease-free water. Nucleic acid concentration and OD_260_/_280_ ratio were measured via NanoDrop. The optimal extraction method was defined as the one that yielded purity-qualified products (OD_260_/_280_ = 1.8–2.0) with the highest nucleic acid concentration. One-way analysis of variance (one-way ANOVA) was used to compare the differences in fungal nucleic acid extraction concentrations under different optimization conditions (e.g., different guanidine hydrochloride concentrations, Triton X-100 concentrations). Fisher’s least significant difference (LSD) method was employed for post hoc multiple comparisons. A *p*-value < 0.05 was considered statistically significant.

### 2.5. Establishment and Optimization of Rapid Detection Methods

#### 2.5.1. Primer and Probe Design

Specific primers and TaqMan probes for *C. neoformans*, *C. albicans*, *A. fumigatus*, and the human internal reference gene were designed in this study. Among them, primers and probes for *C. neoformans* and the human internal reference gene were independently designed and synthesized in our laboratory, while those for *C. albicans* and *A. fumigatus* were designed using AlleleID 7.0 software.

To further verify specificity, all primer and probe sequences were subjected to pairwise sequence alignment and homology analysis using Primer-BLAST on the NCBI database. No cross-reactivity was observed with the human genome or other non-target pathogenic microorganisms, thus eliminating non-specific amplification. All candidate primers and probes that passed specificity screening were synthesized by Sangon Biotech (Shanghai) Co., Ltd.

#### 2.5.2. Optimization of Multiplex qPCR Reaction System and Conditions

Based on the validated primers and probes, a single-tube triplex qPCR detection system was established. Key reaction parameters were optimized using a single-factor variable method on an automated medical PCR analysis system. The optimization factors, gradient settings, and experimental design are summarized in Table 2.

Primer and probe concentrations: with other conditions fixed, primer concentrations were set at five gradients (2, 4, 6, 8, and 10 pmol/system), and probe concentrations were set at six gradients (1, 2, 3, 4, 5, and 6 pmol/system). The optimal combination was determined according to fluorescence intensity and amplification efficiency.

Magnesium ion (Mg^2+^) concentration: five gradients (0, 2, 4, 6, and 8 mM) were tested to evaluate the effect of Mg^2+^ on polymerase activity and amplification specificity, and the optimal concentration was selected accordingly.

Annealing temperature: five gradients (54, 56, 58, 60, and 62 °C) were set using a temperature gradient function. The optimal annealing temperature was determined based on the lowest Ct value and absence of non-specific amplification.

All optimization experiments were performed in triplicate to ensure reliability and reproducibility.

#### 2.5.3. Performance Validation of the Multiplex qPCR System

##### Sensitivity Validation of Singleplex qPCR

Under the optimized singleplex qPCR conditions, serial concentration gradients of standard samples for the target strains (*C. neoformans*, *C. albicans*, and *A. fumigatus*) were tested, with three parallel replicates per gradient. CT values were recorded for each concentration, and the limit of detection (LOD) for each target fungus was determined based on the linear relationship between CT values and standard sample concentrations, thereby evaluating the detection sensitivity of the singleplex qPCR system.

##### Specificity Validation

To verify the specificity of the established detection system, genomic DNAs from closely related fungal species and common clinical pathogens were used as non-target templates for cross-reactivity testing. Tested strains and samples included (but were not limited to): *Candida parapsilosis (C. parapsilosis), Candida tropicalis (C. tropicalis), Candida glabrata (C. glabrata)* (closely related to *Candida* spp.), *Aspergillus niger* (closely related to *Aspergillus* species), *Mycobacterium tuberculosis* (*M. tuberculosis)* (a common clinical pathogen), and human genomic DNA. Both target and non-target templates were subjected to amplification, and the products were separated and visualized by agarose gel electrophoresis. Specificity was comprehensively evaluated by observing the presence of target-specific bands and the absence of non-specific bands, confirming no potential for non-specific amplification.

##### Sensitivity and Repeatability of the Multiplex qPCR System

To assess the sensitivity of the multiplex detection system, mixed standard templates were prepared by combining three target strains at three concentration gradients (1 × 10^1^–1 × 10^8^ CFU/mL) in a 1:1:1 ratio. After multiplex qPCR testing, standard curves were generated with the log_10_ of template copy number as the x-axis and CT value as the y-axis, and linear regression equations were established for each target.

For repeatability validation, intra-assay and inter-assay precision were evaluated. Intra-assay precision was determined by analyzing each sample in three parallel reaction wells, while inter-assay precision was assessed by repeating the experiment three times independently. The coefficient of variation (CV) for both intra- and inter-assay was calculated using the formula: CV = (standard deviation [SD]/mean value [X]) × 100%.

### 2.6. Clinical Application of the Developed Method

#### 2.6.1. Collection of Clinical Sputum Samples

Patients meeting the study’s inclusion criteria were enrolled, and sputum samples were collected in the early morning under fasting conditions (no food or water intake for a specified period prior to collection). Before sampling, participants were instructed to perform three deep breathing cycles followed by forceful coughing to expel sputum from the lower respiratory tract, which was directly collected into sterile tubes. To ensure sample integrity, all collected specimens were transported to the laboratory within 2 h for immediate downstream processing.

#### 2.6.2. Clinical Sample Validation of the Developed Method

To evaluate the clinical applicability of the developed extraction method and single-tube triplex qPCR assay, clinical sputum samples from the hospital were used for methodological validation. The specific procedures are described as follows:

Sample Selection: Clinical sputum samples with confirmed positive/negative results via standard culture-based identification were selected. The sample set included both target fungal-positive and -negative cases to ensure adequate representation and minimize selection bias.

Blinded Testing: Selected clinical samples were processed following a double-blind protocol (i.e., culture identification results were masked from laboratory operators to eliminate subjective bias). Nucleic acids were extracted from each sample using the established high-salt extraction method optimized in this study, followed by analysis via the in-house developed single-tube triplex qPCR assay. Quantitative PCR results, including CT values and positive/negative calls, were systematically documented in a standardized dataset.

Result Comparison and Analysis: The qPCR results were cross-validated against the hospital’s standard culture identification reports. The overall concordance rate was calculated to assess the consistency between the in-house qPCR assay and routine clinical diagnostic methods, providing foundational data support for its potential clinical application.

## 3. Results

### 3.1. Optimization Results of the Developed Fungal Nucleic Acid Extraction Methods

#### 3.1.1. Single-Factor Optimization Results of the Self-Developed High-Salt Lysis Method

A single-factor gradient design was employed to optimize key parameters of the lysis buffer, including guanidine hydrochloride concentration, isopropanol volume fraction, Triton X-100 volume fraction, and system pH. All experiments were performed in triplicate, and nucleic acid purity (OD_260/280_) was assessed using a NanoDrop spectrophotometer. All experimental grruments Hangzhou Dahua Blue Biotechnology Co., Ltd. (Hangzhou, China) ratios between 1.8 and 2.0, meeting the preset quality criteria. Detailed raw data are presented in Tables S1 and S2.

**Figure 1:**
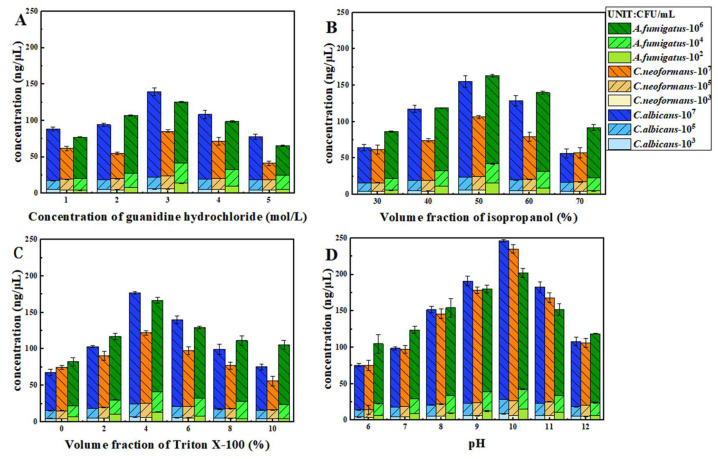
Single-factor optimization results of the self-developed high-salinity lysis method. Nucleic acid extraction efficiency of three pathogenic fungi (*C. albicans, A. fumigatus, and C. neoformans*) under different condition concentrations. Data are presented as the mean ± standard deviation (SD) of three independent experiments (*n* = 3). X-axis: (**A**) Guanidine hydrochloride concentration (mol/L); (**B**) Volume fraction of isopropanol (%); (**C**) Volume fraction of Triton X-100 (%); (**D**) pH. Y-axis: Extracted nucleic acid concentration (ng/μL).

The nucleic acid extraction concentrations of *C. albicans*, *C. neoformans*, and *A. fumigatus* under different conditions are depicted in Figure 1. The optimal extraction efficiency for all three fungi was achieved under the following conditions: 3 mol/L guanidine hydrochloride, 50% isopropanol volume fraction, 4% Triton X-100 volume fraction, and a system pH of 10.

Statistical analyses were performed primarily to provide a preliminary comparison between methods. One-way analysis of variance (one-way ANOVA) was performed separately for the four key extraction factors (guanidine hydrochloride concentration, isopropanol volume fraction, Triton X-100 concentration, and system pH) on the three fungal species (*C. albicans*, *C. neoformans*, and *A. fumigatus*). The results showed that all factors had an extremely significant effect on the nucleic acid extraction concentrations of the three fungal species (all *p* < 0.0001), with the detailed statistical results presented in Table 3. Detailed statistical results for each single-factor optimization are presented in Table S3. Results of Fisher’s least significant difference (LSD) multiple comparison indicated that the optimal level of each factor was consistent for the three fungal species: guanidine hydrochloride concentration at 3 mol/L, isopropanol volume fraction at 50%, Triton X-100 concentration at 4%, and system pH at 10. The nucleic acid extraction concentration under this optimal combination was significantly higher than that at other levels (*p* < 0.05).

#### 3.1.2. Comparative Validation of the Optimized High-Salt Method Versus Commercial Kits

**Figure 2:**
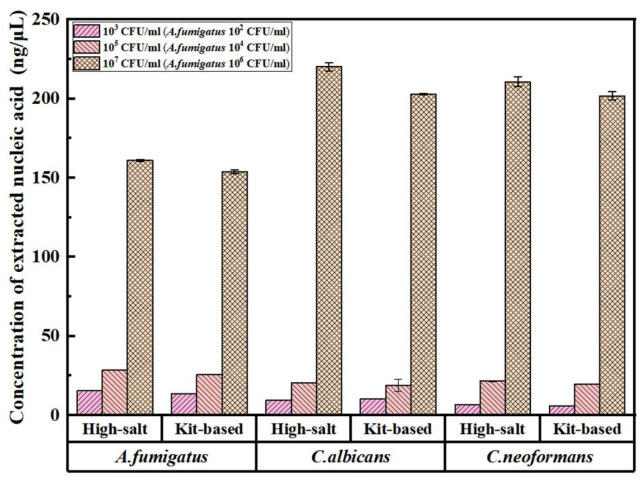
Extraction results of two different fungal nucleic acid extraction methods. Comparison of nucleic acid yields from fungi with different initial concentrations (10^2^–10^7^ CFU/mL) using high-salt extraction and kit-based extraction methods. Data are presented as mean ± SD from three independent replicate experiments (*n* = 3 independent replicate experiments).

**Figure 3:**
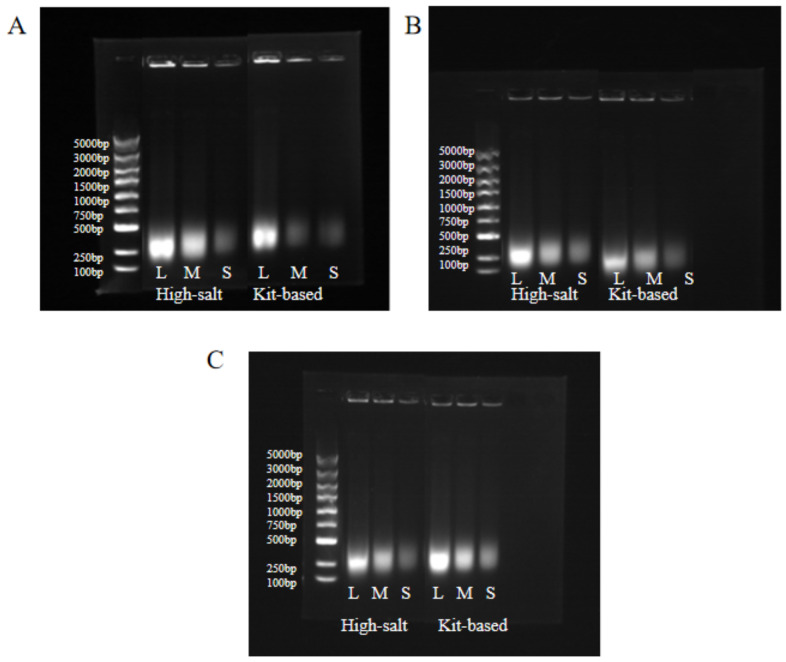
Agarose gel electrophoresis analysis of nucleic acids extracted by the optimized high-salt method and commercial kit. (**A**) *A. fumigatus*, (**B**) *C. albicans*, (**C**) *C. neoformans*. Lanes: M, DNA marker; L, low concentration; M, medium concentration; S, high concentration.

A comparative analysis was conducted between the optimized high-salt method and a commercially available fungal DNA extraction kit (magnetic bead-based) using identical batches of simulated sputum samples. Both extraction protocols were performed in triplicate, and key quality indicators were quantified using a NanoDrop microvolume spectrophotometer. The resultant data are presented in Figure 2. In addition, agarose gel electrophoresis was performed to visually evaluate the integrity and quality of the extracted nucleic acids, as shown in Figure 3.

For *C. albicans*, at a low cell suspension concentration of 10^3^ CFU/mL, the optimized high-salt method yielded a nucleic acid concentration of 9.39 ng/μL, while the commercial kit produced a concentration of 10.32 ng/μL; at a medium concentration of 10^5^ CFU/mL, the high-salt method gave 20.32 ng/μL, compared to 18.79 ng/μL from the commercial kit; at a high concentration of 10^7^ CFU/mL, the high-salt method had a nucleic acid concentration of 220.18 ng/μL, and the commercial kit had 202.85 ng/μL.

For *C. neoformans*, from a low fungal suspension of 10^3^ CFU/mL, the high-salt method yielded a nucleic acid concentration of 6.51 ng/μL, whereas the commercial kit yielded 5.63 ng/μL. At a medium concentration of 10^5^ CFU/mL, the high-salt method gave 21.53 ng/μL, compared with 19.74 ng/μL for the commercial kit; At a high concentration of 10^7^ CFU/mL, the high-salt method achieved 210.76 ng/μL, while the commercial kit reached 201.82 ng/μL.

For *A. fumigatus*, at a low fungal suspension concentration of 10^2^ CFU/mL, the high-salt method yielded 15.43 ng/μL, and the commercial kit had 13.64 ng/μL; at a medium concentration of 10^4^ CFU/mL, the high-salt method gave 28.41 ng/μL, compared to 25.52 ng/μL from the commercial kit; at a high concentration of 10^6^ CFU/mL, the high-salt method had 160.75 ng/μL, while the commercial kit had 153.82 ng/μL.

To statistically assess whether the observed differences in nucleic acid extraction concentrations between the two methods were significant, independent-samples *t*-tests were performed with three parallel replicate extractions for each method. Subsequent experiments will increase the number of replicate tests to further improve the statistical robustness of the results. The results are summarized in Table 4.

For *C. albicans*, at 10^3^ CFU/mL and 10^5^ CFU/mL, the *p*-values were <0.05, indicating significant differences in extraction concentrations between the two methods; at 10^7^ CFU/mL, the *p*-value (0.1726) > 0.05, so no significant difference was observed. For *C. neoformans*, at 10^3^ CFU/mL and 10^5^ CFU/mL, *p*-values were <0.05 (significant differences); at 10^7^ CFU/mL, *p*-value (0.4752) > 0.05 (no significant difference). For *A. fumigatus*, at all tested concentrations (10^2^, 10^4^, 10^6^ CFU/mL), *p*-values were <0.05 (with 10^4^ CFU/mL showing *p* < 0.0001, an extremely significant difference), confirming significant ad-vantages of the optimized high-salt method.

In summary, the optimized high-salt lysis method demonstrates nucleic acid ex-traction performance either superior to or comparable with that of the selected domestic magnetic bead-based commercial kit, while consistently ensuring high-purity fungal nucleic acid (OD_260/280_ = 1.8–2.0). The comparison with international mainstream fungal nucleic acid extraction kits (e.g., Qiagen, Thermo Fisher, Zymo) has not been completed in this study, and follow-up experiments will supplement this comparison to further verify the international superiority of the established method. The enhancement in ex-traction efficiency is most pronounced for *C. neoformans*, particularly advantageous for high-concentration *C. albicans*, and yields stable concentration improvements for *A. fumigatus*. The specific experimental data are presented in Tables S4 and S5.

### 3.2. Establishment and Optimization Results of Rapid Detection Methods

#### 3.2.1. Primer and Probe Design Results

To establish a multiplex real-time qPCR detection system, this study referenced target sequences reported in domestic and international literature. It should be emphasized that the primer and probe sequences differ significantly among the three fungal species. This is mainly attributed to the distinct species-specific conserved target genes of different pathogenic fungi, including the internal transcribed spacer (ITS) region, 18S rRNA, 28S rRNA, and other species-specific gene fragments. The base sequences of these conserved regions vary greatly among different fungal species, which form the molecular basis for specific identification. Therefore, in order to ensure high specificity and no cross-amplification among the three fungi, primers and probes must be designed separately for the unique conserved sequence of each species. Building upon previously established singleplex real-time qPCR detection systems for fungi, we constructed species-specific real-time qPCR assays using probes labeled with distinct fluorophores. The detailed sequences of primers and probes are presented in Table 5. All primers and TaqMan fluorescent probes used in the experiment were synthesized by Shanghai Shenggong Bioengineering Co., Ltd., (Shanghai, China). Synthesized primers and probes were centrifuged briefly and diluted to a stock concentration of 0.1 mM using TE buffer according to the manufacturer’s instructions. Aliquots were stored at 4 °C. Due to the photodegradable nature of fluorescent probes, strict light protection was maintained during storage and handling.

#### 3.2.2. Optimization Results of Multiplex qPCR Reaction Conditions

**Figure 4:**
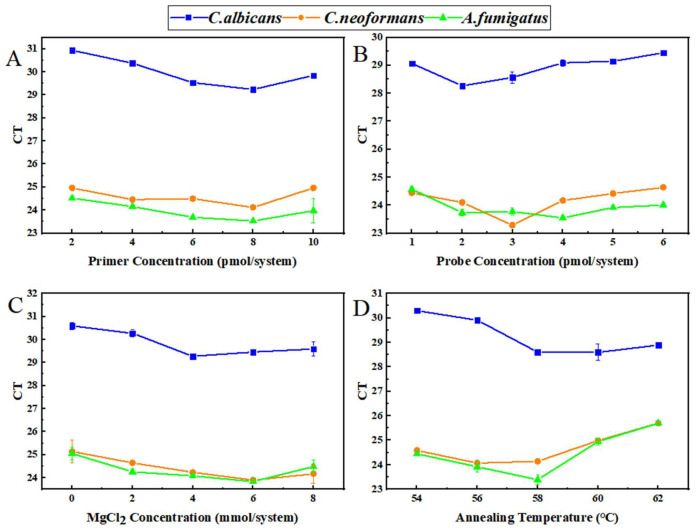
Optimization Results of Multiple qPCR Reaction Conditions. CT values of three pathogenic fungi in multiplex qPCR under different concentration conditions. Data are presented as the mean ± standard deviation of three independent experiments (*n* = 3 independent replicate experiments). X-axis: (**A**) Primer concentration (pmol/system); (**B**) Probe concentration (pmol/system); (**C**) MgCl_2_ concentration (mmol/system); (**D**) Annealing temperature (℃). Y-axis: CT value. *C. albicans* is represented by grey solid lines and squares, *C. neoformans* by orange solid lines and circles, and *A. fumigatus* by blue solid lines and triangles.

To elucidate the effects of primer concentration, probe concentration, MgCl_2_ concentration, and annealing temperature on the amplification efficiency of *C. albicans*, *C. neoformans*, and *A. fumigatus*, systematic optimization of each reaction parameter was performed. CT values served as the primary evaluation index to screen for optimal multiplex amplification parameters. Systematic optimization of primer concentration, probe concentration, MgCl_2_ concentration, and annealing temperature was carried out. When the primer concentration was set at 8 pmol per reaction, the CT values of *C. albicans*, *C. neoformans*, and *A. fumigatus* attained relatively optimal levels. The optimal probe concentrations were determined to be 2 pmol/system for *C. albicans*, 3 pmol/system for *C. neoformans*, and 4 pmol/system for *A. fumigatus*. The optimal MgCl_2_ concentration was 4 mmol/system for *C. albicans* and 6 mmol/system for both *C. neoformans* and *A. fumigatus*. An annealing temperature of approximately 58°C was identified as the optimal temperature for multiplex co-detection. The results are shown in Figure 4, with detailed data provided in Table S6.

Statistical analyses were performed primarily to provide a preliminary comparison between methods. One-way ANOVA was performed on the Ct values under different primer/probe concentrations, Mg^2+^ concentrations and annealing temperatures. The results showed that primer concentrations, probe concentrations, Mg^2+^ concentrations and annealing temperatures all had extremely significant effects on the Ct values of multiplex qPCR amplification for the three pathogenic fungi (*p* < 0.001). Specifically, the F-values for primer concentration ranged from 8.078 to 159.10, for probe concentration ranged from 13.31 to 45.53, for Mg^2+^ concentration ranged from 8.193 to 27.46, and for annealing temperature ranged from 51.55 to 104.60. Detailed statistical results are presented in Table 6. Fisher’s LSD post hoc test verified that 8 pmol per system of primer concentration and 58 °C annealing temperature were the optimal reaction conditions for *C. albicans* and *A. fumigatus* amplification, while 56 °C was optimal for *C. neoformans*. The Ct values under these conditions were significantly lower than those at other gradient levels (*p* < 0.05), with the optimal amplification efficiency and specificity.

#### 3.2.3. Performance Validation Results of the Multiplex qPCR System

##### Sensitivity Results of Singleplex qPCR

**Figure 5:**
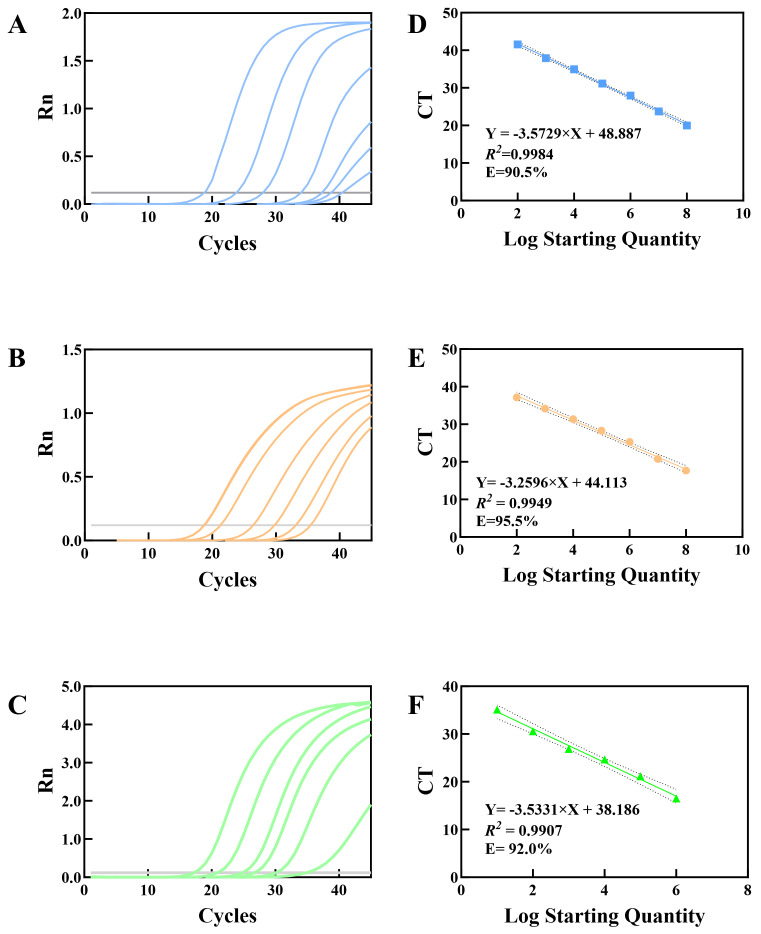
Singleplex qPCR Amplification Curves and Standard Curves for *C. albicans* (**A**), *C. neoformans* (**B**) and *A. fumigatus* (**C**). (**A**–**C**) Amplification curves of *C. albicans*, *C. neoformans*, and *A. fumigatus* at different initial template concentrations, respectively; these curves are representative results from three independent replicate experiments. (**D**–**F**) Corresponding standard curves of the three fungi. Data are presented as mean ± SD from three independent replicate experiments (*n* = 3 independent replicate experiments). X-axis: Logarithm of initial template concentration (log_10_ copies/μL); Y-axis: CT value. The linear regression equation, coefficient of determination (*R*^2^), and amplification efficiency (**E**) are labeled in each subfigure.

To evaluate the detection efficiency of the singleplex qPCR system for target fungi, genomic DNA templates of the three species were prepared at concentration gradients (1 × 10^1^–1 × 10^8^ CFU/mL) for qPCR amplification, and standard curves were constructed. CT values from qPCR amplification were plotted on the y-axis, while the logarithmic values of template concentrations (lg CFU/mL) were plotted on the x-axis. The standard curves for each target fungus were shown in Figure 5. The relevant raw data are presented in Table S7. The regression equation of *C. albicans* was y = −3.5729x + 48.887 (coefficient of determination *R*^2^ = 0.9984), and amplification efficiency was 90.5%. The regression equation of *C. neoformans* was y = −3.2596x + 44.113 (*R*^2^ = 0.995), and amplification efficiency was 95.5%. The regression equation of *A. fumigatus* was y = −3.5331x + 38.186 (*R*^2^ = 0.991), and amplification efficiency was 92.0%. All three standard curves exhibited *R*^2^ values > 0.99, indicating a strong linear correlation between CT values and the logarithm of template concentrations, confirming that the quantitative accuracy of the qPCR system meets experimental requirements. Further analysis of gradient concentration template amplification results determined the detection limits: 1 × 10^3^ CFU/mL for *C. albicans*, 1 × 10^2^ CFU/mL for *C. neoformans*, and 1 × 10^1^ CFU/mL for *A. fumigatus*. These results demonstrate the highest sensitivity for *A. fumigatus* and strong detection capabilities for all target fungi.

##### Specificity Verification Results

**Figure 6:**
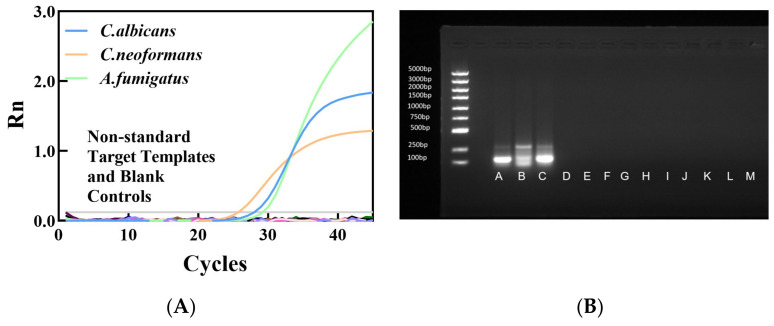
Specificity Verification Amplification Results (**A**) and Gel Electrophoresis Results (**B**) A: *C. albicans*; B: *C. neoformans*; C: *A. fumigatus*; D: *A.niger*; E: *A.terreus*; F: *M.tuberculosis*; G: *E.coli*; H: *S. aureus;* I: *C.tropical*; J: *C.glabrata*; K: *C.krusei;* L: *B.subtilis*; M: *Human*.

The specificity of the multiplex qPCR system was evaluated, and representative results are shown in Figure 6. As illustrated in Figure 6A, positive amplification signals (specific bands) were detected only in reactions containing the target fungal DNA, whereas no amplification was observed for non-target microorganisms. Figure 6B shows representative bands from three independent replicate experiments. The left side of the gel indicates the DNA marker sizes (bp: 5000, 3000, 2000, 1500, 1000, 750, 500, 250, 100). Lane designations correspond to the strains described in Figure 6B. These results are consistent with our experimental expectations, confirming that the established multiplex qPCR system exhibits high specificity and no cross-amplification with other clinically common fungi or bacteria.

##### Sensitivity and Reproducibility Results of the Multiplex qPCR System

**Figure 7:**
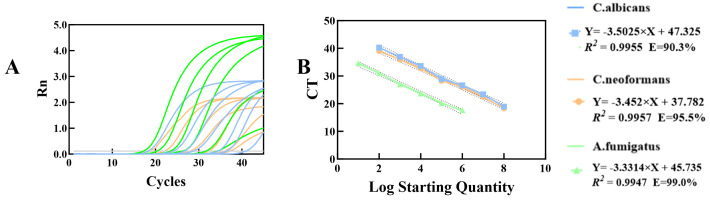
Analysis of Amplification Sensitivity and Standard Curve for Multiplex qPCR (**A**) Amplification curves of *C. albicans*, *C. neoformans*, and *A. fumigatus* at gradient template concentrations in multiplex qPCR, which are representative results from three independent replicate experiments. (**B**) Corresponding standard curves of the three fungi. Data are presented as mean ± SD from three independent replicate experiments (*n* = 3 independent replicate experiments).

To assess the sensitivity of the multiplex qPCR system, fluorescent quantitative detection was performed on mixed cultures of the three target strains at concentration gradients (1 × 10^1^–1 × 10^8^ CFU/mL). Multiplex standard curves were constructed based on the obtained CT values to calculate the amplification efficiency for each target. Standard curve analysis revealed the following results: the regression equation of *C. albicans* was y = −3.5025x + 47.325 (coefficient of determination *R*^2^ = 0.9955), and amplification efficiency was 90.3%; the regression equation of *C. neoformans* was y = −3.3314x + 45.735 (*R*^2^ = 0.9947), and amplification efficiency was 99.0%; the regression equation of *A. fumigatus* was y = −3.452x + 37.782 (*R*^2^ = 0.9916), and amplification efficiency was 95.5%. All three standard curves exhibited *R*^2^ values > 0.99, indicating excellent linear correlation between CT values and the logarithm of template concentrations. The detection limits of the multiplex qPCR system for each target were consistent with those of the singleplex assay: 1 × 10^3^ CFU/mL for *C. albicans*, 1 × 10^2^ CFU/mL for *C. neoformans*, and 1 × 10^1^ CFU/mL for *A. fumigatus*. The standard curves were shown in Figure 7. The relevant raw data are presented in Table S8.

To evaluate the reproducibility and reliability of the multiplex qPCR system, intra-batch and inter-batch repeatability assays were performed, each in triplicate.

Intra-batch repeatability refers to repeated detection of the same sample within a short period of time, which was designed to evaluate the short-term stability of the established detection system and verify its consistency during immediate and continuous testing. Inter-batch repeatability refers to repeated detection of the same sputum sample at different time intervals, which was used to assess the standardization and long-term stability of the entire detection system, ensuring that results remain consistent and unaffected by different operating cycles or time intervals.

These two distinct validation strategies were applied to comprehensively evaluate the stability and repeatability of the multiplex qPCR system, confirming its reliable and consistent performance under different experimental scenarios, which is critical for its practical clinical application.

As shown in Table S9, the coefficient of variation (CV) values for each target were as follows:

*C. albicans*: Intra-batch CV = 0.28–2.22%, inter-batch CV = 0.21–2.02%

*C. neoformans*: Intra-batch CV = 0.88–2.94%, inter-batch CV = 0.32–2.35%

*A. fumigatus*: Intra-batch CV = 0.28–2.22%, inter-batch CV = 0.78–2.02%

All CV values were below 5%, which meets the international acceptance criteria for qPCR assays [21]. These results demonstrate that the established multiplex qPCR method possesses excellent reproducibility and reliability.

### 3.3. Clinical Application

#### 3.3.1. Sputum Sample Preprocessing

In the clinical sample validation phase, real patient sputum samples exhibited distinct physical properties compared to the artificial sputum used in preliminary methodological validation. Specifically, clinical sputum samples typically had higher viscosity and were often interspersed with intractable gel-like clumps that cannot be directly dispersed. Untreated impurities of this nature would severely compromise the stability of subsequent experimental systems and the accuracy of detection results. Therefore, a targeted preprocessing step was incorporated prior to the core procedures of the in-house detection method. The preprocessing protocol was as follows: collected patient sputum samples were transferred to sterile centrifuge tubes and subjected to ultrasonic liquefaction. The cavitation effect and mechanical vibrations generated by ultrasound disrupt the mucin network structure in sputum, enabling complete dispersion and breakdown of stubborn gel clumps to form a homogenized sputum preparation. Following preprocessing, the homogenized samples were subjected to subsequent nucleic acid extraction, qPCR reaction, and detection procedures in accordance with the standard operating workflow of the in-house method.

#### 3.3.2. Clinical Validation Results

Clinical sample validation was performed to evaluate the performance of the established method. A total of 14 sputum samples were collected from patients with suspected respiratory fungal infections, and the results were compared with those of conventional fungal culture, which is the current gold standard in clinical practice. The overall agreement rate between our method and the hospital’s conventional culture was 92.85%, with detailed comparative data presented in Table 7.

For the consistency analysis of detection methods, the gold standard was used as the reference. Among the 14 clinical samples evaluated by qPCR, there were 6 true negatives, 1 false positive, 0 false negatives, and 7 true positives when compared with the gold standard. The Cohen’s kappa coefficient was calculated to assess inter-method reliability:

- Observed agreement $P_{o}\;=\;\frac{+7}{14}\;\approx \;0.9286$
- Agreement by chance $P_{C}\;=\;\frac{7\;\times \;6\;+\;7\;\times \;8}{14^{2}}\;=\;0.5$
- Final K=$\frac{P_{o}\;-\;P_{C}}{1\;-{\;P}_{C}}\;\approx \;0.857\;>\;0.8$, indicating a high degree of consistency (K > 0.8).

## 4. Discussion

### 4.1. Key Findings and Mechanistic Interpretation

The present study successfully established a mechanism-guided integrated diagnostic system for pulmonary IFIs, consisting of an optimized high-salt lysis method and a single-tube multiplex qPCR assay.

The optimized high-salt lysis method demonstrated superior performance in nucleic acid extraction from complex sputum matrices. The single-factor optimization identified 3 mol/L guanidine hydrochloride, 50% isopropanol, 4% Triton X-100, and pH 10 as the optimal conditions. This combination is mechanistically sound: guanidine hydrochloride effectively denatures proteins and disrupts the fungal cell wall/capsule structure; Triton X-100 enhances membrane permeability by solubilizing lipid components; and the alkaline environment (pH 10) weakens the hydrogen and ionic bonds within the cell wall, facilitating the penetration of lysis reagents. The comparative study confirmed that this method outperformed the commercial kit, especially at low to medium concentrations. This superiority is particularly notable for *A. fumigatus* and *C. neoformans*, which possess unique cell wall structures (porous filaments and polysaccharide capsules, respectively) that are often resistant to standard lysis protocols. The non-significant difference at high concentrations (10^7^ CFU/mL) likely reflects a saturation effect of nucleic acid yield, where both methods are sufficient to lyse the abundant fungal cells.

The developed multiplex qPCR system exhibited high sensitivity, specificity, and reproducibility. The design of species-specific primers and probes targeting conserved genomic regions (ITS, rRNA) ensured no cross-reactivity with non-target pathogens or human DNA, which is critical for avoiding false positives in clinical diagnosis. The LODs (10^1^–10^3^ CFU/mL) are comparable to or better than those reported in previous studies, indicating the system’s ability to detect low-burden infections. The intra-batch and inter-batch CV values of <5% confirm the system’s robustness and reliability, supporting its potential for clinical application.

Clinical validation demonstrated promising diagnostic potential. Despite the small sample size (*N* = 14), the high consistency with the gold standard (Cohen’s kappa coefficient = 0.857, indicating substantial agreement) validates the proof-of-concept. The single false positive result (Patient J) may be attributed to the higher sensitivity of qPCR, which can detect non-viable fungal DNA or early colonization that precedes culture positivity—a known advantage of molecular diagnostics over traditional culture methods.

### 4.2. Comparison with Previous Studies

In recent years, various molecular diagnostic methods for fungal detection have been developed. Most existing studies focus either on nucleic acid extraction or qPCR establishment alone, while few provide an integrated system from sample processing to detection. Compared with those studies, our work highlights two major innovations. First, we performed mechanism-guided optimization of the lysis system according to the distinct cell wall structures of different fungi, rather than blind parameter screening. This approach improves the scientificity and repeatability of method development. Second, the integrated workflow combines high-efficiency extraction and multiplex qPCR, enabling completion of detection within 2 h, which is much faster than traditional fungal culture that usually takes days. Several studies have reported multiplex qPCR for fungal detection; however, many used complex reaction systems or lacked rigorous validation in clinically representative matrices such as sputum. In contrast, our system was systematically optimized and validated in simulated and real clinical sputum samples, with complete analytical performance evaluation including sensitivity, specificity, reproducibility, and clinical preliminary verification. Therefore, the present study provides a more practical and clinically oriented diagnostic tool.

### 4.3. Limitations

This study has several important limitations that should be acknowledged.

(1) Simulated sputum system limitations: the simulated sputum used in method optimization was formulated using pectin and CMC-Na, which only mimicked the viscosity of clinical sputum [22], but did not incorporate bioactive components such as immune cells or co-existing pathogenic bacteria [23]. Consequently, the interference of these components on nucleic acid extraction and qPCR detection could not be fully evaluated, which may have led to an overestimation of the method’s clinical robustness. Furthermore, validation of the simulated system was limited to viscosity measurements, without systematic analysis of its similarity to real sputum in terms of viscoelasticity, chemical microenvironment, or other biophysical characteristics. The relationship between matrix composition and different pathological types of sputum was also not explored. In addition, the validation was based on a single simulated system, lacking external validation with multi-center real sputum samples, which makes it difficult to assess the method’s generalizability and stability across diverse clinical settings.
(2) Methodological comparison limitations: the established high-salt lysis method was only benchmarked against one domestic magnetic bead-based commercial kit. No direct comparison was performed with internationally recognized mainstream kits (e.g., Qiagen, Thermo Fisher), which reduces the international comparability and persuasiveness of the experimental results. Future studies should include head-to-head comparisons with these gold-standard extraction kits to strengthen the evidence base.
(3) Although the number of clinical samples was limited (*n* = 14), the present study was designed as a proof-of-concept validation. The results demonstrated a good agreement between the developed qPCR assay and conventional culture methods (Kappa > 0.85). However, due to the limited sample size, the diagnostic performance indicators should be interpreted cautiously. Future studies with larger sample sizes and multi-center validation will be necessary to further confirm the robustness and clinical applicability of this method.

### 4.4. Future Directions

Future improvements should focus on several key aspects: constructing a more physiologically relevant simulated sputum system that incorporates bioactive components, or directly using real sputum for validation [24]; expanding the evaluation dimensions of the simulated system and establishing standardized formulations corresponding to different pathological sputum types; incorporating multi-center clinical data to validate the clinical value and generalizability of the method; conducting head-to-head comparisons with internationally recognized commercial extraction kits to strengthen the methodological rigor. Only through these improvements can the effectiveness and practicality of the method in clinical practice be evaluated more comprehensively and accurately, providing more reliable technical support for the diagnosis of pulmonary fungal infections.

## 5. Conclusions

Based on a strategy of mechanism-guided optimization driven by fungal cell wall architecture, this study established an integrated diagnostic system for three WHO critical-priority respiratory pathogenic fungi, consisting of a customized nucleic acid extraction protocol and a single-tube triple qPCR assay. This approach abandons the traditional blind parameter screening and realizes targeted optimization of the lysis system according to the distinct structural characteristics of fungal pathogens, which represents the core innovation of this work. The established high-salt lysis method, guided by cell wall mechanism, is more scientific and reproducible compared with conventional blind optimization strategies, and provides a novel technical paradigm for efficient fungal nucleic acid extraction from complex clinical samples such as sputum. The integrated system demonstrates superior performance in terms of rapidity, sensitivity, specificity, and stability, enabling detection within 2 h—far faster than traditional fungal culture—and shows excellent concordance with conventional methods in clinical validation, with enhanced sensitivity for low-load infections. This work not only provides a reliable technical support for rapid and accurate diagnosis of pulmonary fungal infections, thereby advancing clinical diagnostic efficiency and guiding precise antifungal therapy, but also offers a mechanism-driven framework for the development of diagnostic tools targeting other pathogens with complex cell structures. Future directions include large-scale multi-center validation and automation integration to further improve workflow efficiency and clinical applicability.

## Figures and Tables

**Table 1:** Factors and gradient settings for the optimization of fungal nucleic acid extraction.

Optimization Factor	Gradient Settings
Guanidine hydrochloride concentration	1, 2, 3, 4, 5 mol/L
Isopropanol volume fraction	30%, 40%, 50%, 60%, 70%
Triton X-100 volume fraction	0%, 2%, 4%, 6%, 8%, 10%
System pH	6, 7, 8, 9, 10, 11, 12

**Table 2:** Optimization factors and gradient settings for multiplex qPCR system.

Optimization Factor	Gradient Settings
Primer concentration	2, 4, 6, 8, 10 pmol/system
Probe concentration	1, 2, 3, 4, 5, 6 pmol/system
Mg^2+^ concentration	0, 2, 4, 6, 8 mM
Annealing temperature	54, 56, 58, 60, 62 °C

**Table 3:** ANOVA Results of the Effects of Different Optimization Factors on Fungal Nucleic Acid Extraction Concentration (low concentration).

Fungal Species	Optimization Factor	Number of Levels	F-Value	*p*-Value	Optimum Level
*C. albicans*	Concentration of Guanidine Hydrochloride (mol/L)	5	59.01	<0.0001	3 mol/L
	Volume fraction of isopropanol (%)	5	220.3	<0.0001	50%
	Volume fraction of Triton X-100 (%)	6	334.8	<0.0001	4%
	System pH	7	1344	<0.0001	10
*C. neoformans*	Concentration of Guanidine Hydrochloride (mol/L)	5	69.82	<0.0001	3 mol/L
	Volume fraction of isopropanol (%)	5	224.6	<0.0001	50%
	Volume fraction of Triton X-100 (%)	6	246.5	<0.0001	4%
	System pH	7	315.1	<0.0001	10
*A. fumigatus*	Concentration of Guanidine Hydrochloride (mol/L)	5	3908	<0.0001	3 mol/L
	Volume fraction of isopropanol (%)	5	7226	<0.0001	50%
	Volume fraction of Triton X-100 (%)	6	672.6	<0.0001	4%
	System pH	7	1262	<0.0001	10

**Table 4:** *t*-test Comparison: High Salt Method vs. Commercial Kit.

	CFU/mL	High-Salt (ng/μL)	Kit-Based (ng/μL)	t-Value	*p*-Value	Significance
*C. albicans*	10^3	9.39	10.32	−7.26	0.0098	+
	10^5	20.20	18.76	9.02	0.0064	+
	10^7	220.38	202.85	1.94	0.1726	−
*C. neoformans*	10^3	6.51	5.63	17.86	0.0017	+
	10^5	20.59	19.74	8.36	0.0072	+
	10^7	225.03	210.82	−0.85	0.4758	−
*A. fumigatus*	10^2	15.43	13.64	22.54	0.0011	+
	10^4	28.41	25.52	111.93	<0.0001	+
	10^6	160.95	153.82	11.26	0.0042	+

Table 4 Notes: “+” indicates a significant difference (*p* < 0.05) in nucleic acid extraction concentrations between the optimized high-salt method and the commercial kit; “−” indicates no significant difference (*p* > 0.05).

**Table 5:** Primer and TaqMan Probe Sequences Used in qPCR Detection in This Study.

	Oligonucleotide (5′-3′)		Probe Labeling
*C. albicans*	F	TTGTGTTTGGTAGTGAGTGATACTC	
	R	CGAGCAGCAGATTAATAGAGAAGC	
	p	CGTTCGTGTCCCACATATTGATATGGC	ROX/BHQ2
*C. neoformans*	F	GTTGGACTTGGATTTGGGTGTT	
	R	GGCCCAGCGAAACTTATTACG	
	p	CGACCTGCAAAGGACGTCGGCTCT	VIC/BHQ1
*A. fumigatus*	F	AGTGGGACCGCAAGTGACAT	
	R	CCCACGATCCATCTTCGATT	
	p	CATGTGTTTGGCCTGC	FAM/MGB
globin	F	ATTGGACAGCAAGAAAGCGA	
	R	CTGCCTATCAGAAAGTGGTGG	
	p	CTGGTGTGGCTAATGCCCTGGC	CY5/BHQ2

**Table 6:** ANOVA Results of the Effects of Different qPCR Reaction Conditions on Ct Values.

Fungal Species	Optimization Factor	Number of Levels	F-Value	*p*-Value	Optimum Level
*C. albicans*	Primer concentration (pmol/system)	5	342.7	<0.0001	8
	Probe concentration (pmol/system)	6	45.53	<0.0001	2
	Mg^2+^ concentration (mmol/system)	6	27.46	<0.0001	4
	Annealing Temperature (℃)	7	66.70	<0.0001	58
*C. neoformans*	Primer concentration (pmol/system)	5	159.1	<0.0001	8
	Probe concentration (pmol/system)	6	43.25	<0.0001	3
	Mg^2+^ concentration (mmol/system)	6	8.193	<0.0001	6
	Annealing Temperature (℃)	7	515.5	<0.0001	56
*A. fumigatus*	Primer concentration (pmol/system)	5	8.078	<0.0001	8
	Probe concentration (pmol/system)	6	13.31	<0.0001	4
	Mg^2+^ concentration (mmol/system)	6	19.58	<0.0001	6
	Annealing Temperature (℃)	7	104.6	<0.0001	58

**Table 7:** Comparison of detection results between the multiplex qPCR method and conventional fungal culture in clinical sputum samples (*n* = 14).

Patients	Hospital’s Results	Our Results
A	+	+
B	+	+
C	+	+
D	+	+
E	+	+
F	+	+
G	+	+
H	−	−
I	−	−
J	−	+
K	−	−
L	−	−
M	−	−
N	−	−

## Data Availability

Data will be made available on request.

## Supplementary Materials

The following supporting information can be downloaded at https://www.mdpi.com/article/10.3390/jof12040252/s1, Table S1: Single-Factor Optimization Results of the Self-Developed High-Salinity Lysate Method (Concentration); Table S2: Single-Factor Optimization Results of the Self-Developed High-Salt Lysis Method (Purity: OD_260_/_280_); Table S3: ANOVA Results of the Effects of Different Optimization Factors on Fungal Nucleic Acid Extraction Concentration (medium and high concentration) Table S4: Extraction Results of Two Different Fungal Nucleic Acid Extraction Methods (Concentration); Table S5: Extraction results of two different fungal nucleic acid extraction methods (purity); Table S6: Optimization Results of Multiple qPCR Reaction Conditions; Table S7: Sensitivity Results of Single qPCR Reaction System; Table S8: Sensitivity and Reproducibility Results of the Multiplex qPCR Reaction System; Table S9: Sensitivity and Reproducibility Results of the Multiplex qPCR Reaction System.
