# Supplementary material for: An Efficient Workflow for Fungal Nucleic Acid Detection in Sputum
Source: J Fungi (Basel). 2026 Mar 31;12(4):252. doi: 10.3390/jof12040252 (PMC13117515; doi:10.3390/jof12040252)
Supplement: Supplementary file 1 [file jof-12-00252-s001.zip › jof-4178191-supplementary.pdf]

Table S1. Single-Factor Optimization Results of the Self-Developed High-Salinity Lysate Method (Concentration)

		C.albicans																	
	(CFU/ml)	10^3						10^5						10^7					
		Concentration (ng/μL)			Mean	SD	CV	Concentration (ng/μL)			Mean	SD	CV	Concentration (ng/μL)			Mean	SD	CV
Concentration of Guanidine Hydrochloride (mol/L)	1	5.33	5.18	5.29	5.27	0.08	1.47%	12.35	12.82	12.93	12.70	0.31	2.43%	71.41	72.53	69.81	71.25	1.37	1.92%
	2	5.24	5.37	5.12	5.24	0.13	2.38%	13.25	13.13	13.33	13.24	0.10	0.76%	76.64	73.24	77.33	75.74	2.19	2.89%
	3	6.05	5.92	6.08	6.02	0.09	1.41%	16.55	16.45	16.63	16.54	0.09	0.55%	112.15	116.46	112.82	113.81	2.32	2.04%
	4	5.22	5.25	5.38	5.28	0.09	1.61%	14.05	13.92	14.13	14.03	0.11	0.76%	91.15	88.43	86.45	88.68	2.36	2.66%
	5	4.82	4.93	4.97	4.91	0.08	1.58%	13.55	13.42	13.63	13.53	0.11	0.78%	63.23	65.52	64.17	64.31	1.15	1.79%
Volume fraction of isopropanol (%)	30	4.85	4.76	4.89	4.83	0.07	1.38%	11.25	11.12	11.33	11.23	0.11	0.94%	69.32	68.31	71.94	69.86	1.87	2.68%
	40	5.52	5.43	5.58	5.51	0.08	1.37%	13.85	13.72	13.98	13.85	0.13	0.94%	100.16	98.63	102.16	100.32	1.77	1.76%
	50	6.23	6.15	6.29	6.22	0.07	1.13%	17.35	17.22	17.33	17.30	0.07	0.40%	131.21	128.96	130.43	130.20	1.14	0.88%
	60	5.42	5.35	5.48	5.42	0.07	1.20%	14.55	14.42	14.63	14.53	0.11	0.73%	113.85	111.23	111.52	112.20	1.44	1.28%
	70	4.72	4.63	4.78	4.71	0.08	1.60%	12.05	11.92	12.42	12.13	0.26	2.14%	74.24	76.96	72.37	74.52	2.31	3.10%
Volume fraction of Triton X-100 (%)	0	4.71	4.53	4.58	4.61	0.09	2.02%	10.53	10.41	10.63	10.52	0.11	1.05%	51.23	51.51	49.87	50.87	0.88	1.72%
	2	5.34	5.28	5.35	5.32	0.04	0.71%	13.13	12.95	13.18	13.09	0.12	0.92%	86.42	83.53	85.24	85.06	1.45	1.71%
	4	6.53	6.42	6.59	6.51	0.09	1.32%	18.26	17.92	18.15	18.11	0.17	0.96%	153.02	154.29	150.85	152.72	1.74	1.14%
	6	5.67	5.57	5.62	5.62	0.05	0.89%	14.70	14.74	14.96	14.80	0.14	0.95%	122.78	121.90	122.36	122.35	0.44	0.36%
	8	5.02	4.93	5.08	5.01	0.08	1.51%	12.24	12.16	12.24	12.21	0.05	0.38%	84.66	82.87	83.23	83.59	0.95	1.13%
	10	4.62	4.53	4.62	4.59	0.05	1.13%	11.46	10.98	11.26	11.23	0.24	2.15%	61.25	58.92	62.65	60.94	1.88	3.09%
pH	6	4.34	4.15	4.25	4.25	0.10	2.24%	9.82	9.61	9.94	9.79	0.17	1.71%	60.83	59.21	61.21	60.42	1.06	1.76%
	7	5.02	4.98	5.03	5.01	0.03	0.53%	12.53	12.42	12.69	12.55	0.14	1.08%	82.56	78.54	82.50	81.20	2.30	2.84%
	8	5.63	5.53	5.68	5.61	0.08	1.36%	15.28	15.12	15.38	15.26	0.13	0.86%	133.57	130.32	133.25	132.38	1.79	1.35%
	9	6.12	6.09	6.17	6.13	0.04	0.66%	17.05	16.96	17.13	17.05	0.09	0.50%	160.62	163.67	164.31	162.87	1.97	1.21%
	10	8.87	8.70	8.89	8.82	0.10	1.18%	19.36	19.41	19.67	19.48	0.17	0.85%	219.13	216.26	218.54	217.98	1.52	0.70%
	11	6.23	6.15	6.24	6.21	0.05	0.79%	16.85	16.75	16.93	16.84	0.09	0.54%	161.28	161.51	166.32	163.04	2.85	1.75%
	12	5.31	5.23	5.32	5.29	0.05	0.93%	13.42	13.02	13.21	13.22	0.20	1.51%	90.17	93.62	94.94	92.91	2.46	2.65%

Table S1. Single-Factor Optimization Results of the Self-Developed High-Salinity Lysate Method (Concentration)

	C.neoformans		
(CFU/ml)	10^3	10^5	10^7

		Concentration（ng/μL）			Mean	SD	CV	Concentration（ng/μL）			Mean	SD	CV	Concentration（ng/μL）			Mean	SD	CV
Concentration of Guanidine Hydrochloride（mol/L）	1	4.75	4.93	4.81	4.83	0.09	1.90%	14.30	14.72	14.45	14.49	0.21	1.47%	32.26	31.53	31.12	31.64	0.58	1.83%
	2	5.12	4.92	5.05	5.03	0.10	2.02%	15.35	14.93	15.12	15.13	0.21	1.39%	46.23	44.16	45.92	45.44	1.12	2.46%
	3	5.95	6.02	6.25	6.07	0.16	2.58%	17.98	17.63	17.85	17.82	0.18	0.99%	62.67	59.47	62.41	61.52	1.78	2.89%
	4	5.32	5.15	5.28	5.25	0.09	1.69%	15.50	15.11	15.31	15.31	0.20	1.27%	47.39	48.85	48.17	48.14	0.73	1.52%
	5	4.58	4.40	4.65	4.54	0.13	2.84%	14.40	14.08	14.25	14.24	0.16	1.12%	26.62	25.28	25.73	25.88	0.68	2.64%
Volume fraction of isopropanol（%）	30	4.30	4.08	4.22	4.20	0.11	2.65%	12.35	11.92	12.14	12.14	0.22	1.77%	49.25	48.89	50.84	49.66	1.04	2.09%
	40	4.88	4.96	4.79	4.88	0.09	1.74%	14.95	14.55	14.73	14.74	0.20	1.36%	57.62	56.47	54.90	56.33	1.37	2.42%
	50	6.20	6.01	6.15	6.12	0.10	1.61%	18.75	18.39	18.57	18.57	0.18	0.97%	83.59	79.54	81.92	81.68	2.04	2.49%
	60	5.35	5.42	5.27	5.35	0.08	1.40%	15.45	15.73	15.26	15.48	0.24	1.53%	61.43	61.18	63.35	61.99	1.19	1.92%
	70	4.32	4.41	4.25	4.33	0.08	1.85%	13.25	12.81	13.25	13.10	0.25	1.94%	42.19	42.37	43.17	42.58	0.52	1.23%
Volume fraction of Triton X-100（%）	0	4.01	4.15	3.95	4.04	0.10	2.54%	11.25	10.81	11.25	11.10	0.25	2.29%	62.24	59.64	60.41	60.76	1.34	2.20%
	2	5.32	5.24	5.33	5.30	0.05	0.93%	14.55	14.17	14.32	14.35	0.19	1.33%	77.75	78.45	75.49	77.23	1.55	2.00%
	4	6.42	6.21	6.35	6.33	0.11	1.69%	19.45	19.32	19.25	19.34	0.10	0.52%	93.41	97.35	99.14	96.63	2.93	3.03%
	6	5.62	5.40	5.55	5.52	0.11	2.03%	15.75	15.34	15.57	15.55	0.21	1.32%	74.54	76.92	73.36	74.94	1.81	2.42%
	8	4.75	4.82	4.65	4.74	0.09	1.80%	13.45	13.21	13.26	13.31	0.13	0.95%	63.42	61.51	60.24	61.72	1.60	2.59%
	10	4.08	4.21	3.98	4.09	0.12	2.82%	12.35	11.95	12.16	12.15	0.20	1.65%	46.72	44.68	45.47	45.62	1.03	2.25%
pH	6	3.88	4.01	3.90	3.93	0.07	1.78%	10.45	10.32	10.29	10.35	0.09	0.82%	65.14	63.14	65.16	64.48	1.16	1.80%
	7	4.82	4.63	4.75	4.73	0.10	2.03%	14.13	13.63	13.86	13.87	0.25	1.80%	83.63	80.54	79.92	81.36	1.99	2.44%
	8	5.54	5.32	5.45	5.44	0.11	2.03%	16.75	16.37	16.54	16.55	0.19	1.15%	126.82	126.51	128.45	127.26	1.04	0.82%
	9	6.12	5.93	6.05	6.03	0.10	1.59%	18.35	17.91	18.14	18.13	0.22	1.21%	158.27	159.73	155.24	157.75	2.29	1.45%
	10	6.72	6.50	6.65	6.62	0.11	1.70%	20.25	19.82	20.12	20.06	0.22	1.10%	214.71	213.90	216.09	214.90	1.11	0.52%
	11	6.22	6.21	6.15	6.19	0.04	0.61%	18.28	19.21	19.15	18.88	0.52	2.76%	145.42	145.25	148.52	146.40	1.84	1.26%
	12	5.35	5.18	5.25	5.26	0.09	1.62%	15.34	15.13	15.25	15.24	0.11	0.69%	90.93	88.34	88.83	89.37	1.38	1.54%

Table S1. Single-Factor Optimization Results of the Self-Developed High-Salinity Lysate Method (Concentration)

		A.fumigatus																	
	（CFU/ml）	10^2						10^4						10^6					
		Concentration（ng/μL）			Mean	SD	CV	Concentration（ng/μL）			Mean	SD	CV	Concentration（ng/μL）			Mean	SD	CV
Concentration of Guanidine	1	4.29	4.38	4.42	4.36	0.07	1.53%	15.98	15.86	16.31	16.05	0.23	1.45%	57.46	56.39	56.53	56.79	0.58	1.02%
	2	8.18	8.36	8.11	8.22	0.13	1.57%	19.46	19.25	19.53	19.41	0.15	0.75%	78.93	78.86	79.99	79.26	0.63	0.80%

Hydrochloride (mol/L)	3	13.95	13.88	14.14	13.99	0.13	0.96%	27.53	27.46	27.69	27.56	0.12	0.43%	83.26	85.19	83.33	83.93	1.09	1.30%
	4	9.72	9.53	9.65	9.63	0.10	1.00%	23.25	23.15	23.32	23.24	0.09	0.37%	66.82	66.75	63.89	65.82	1.67	2.54%
	5	5.86	5.79	5.93	5.86	0.07	1.19%	18.68	18.65	18.75	18.69	0.05	0.27%	40.46	40.39	41.53	40.79	0.64	1.57%
Volume fraction of isopropanol (%)	30	5.84	5.87	5.91	5.87	0.04	0.60%	16.39	16.32	16.56	16.42	0.12	0.75%	64.32	63.75	64.89	64.32	0.57	0.89%
	40	11.52	11.35	11.59	11.49	0.12	1.07%	20.93	20.86	20.99	20.93	0.07	0.31%	86.39	85.82	86.46	86.22	0.35	0.41%
	50	15.88	15.81	15.95	15.88	0.07	0.44%	26.86	26.79	25.93	26.53	0.52	1.95%	123.09	120.02	119.18	120.76	2.06	1.70%
	60	9.15	9.18	9.22	9.18	0.04	0.38%	22.59	22.42	22.66	22.56	0.12	0.55%	109.66	105.49	109.73	108.29	2.43	2.24%
	70	4.93	4.86	5.12	4.97	0.13	2.71%	17.66	17.89	18.03	17.86	0.19	1.05%	73.96	75.85	76.03	75.28	1.15	1.52%
Volume fraction of Triton X-100 (%)	0	6.67	6.68	6.54	6.63	0.08	1.18%	15.46	15.29	15.53	15.43	0.12	0.80%	63.89	65.92	64.06	64.62	1.13	1.74%
	2	10.19	10.32	10.26	10.26	0.07	0.63%	19.93	19.92	20.06	19.97	0.08	0.39%	85.26	86.29	83.53	85.03	1.39	1.64%
	4	13.42	13.15	13.49	13.35	0.18	1.34%	28.16	27.99	28.13	28.09	0.09	0.32%	127.46	122.55	124.73	124.91	2.46	1.97%
	6	10.32	10.84	9.94	8.01	0.12	1.54%	24.56	24.55	24.74	24.62	0.11	0.43%	98.91	97.23	95.16	97.10	1.88	1.93%
	8	7.98	7.91	8.15	4.49	0.04	0.78%	23.82	23.75	23.89	23.82	0.07	0.29%	83.13	88.16	86.20	85.83	2.54	2.95%
	10	4.46	4.49	4.53	4.49	0.04	0.78%	19.09	19.12	19.16	19.12	0.04	0.18%	75.59	77.62	79.66	77.62	2.04	2.62%
pH	6	6.35	6.18	6.32	6.28	0.09	1.44%	16.73	16.46	16.80	16.66	0.18	1.08%	72.39	74.89	75.46	74.25	1.63	2.20%
	7	8.89	8.82	8.96	8.89	0.07	0.79%	20.39	20.32	20.47	20.39	0.08	0.37%	99.93	96.42	95.99	97.45	2.16	2.22%
	8	9.53	9.46	9.67	9.55	0.11	1.12%	24.16	24.49	24.23	24.29	0.17	0.72%	110.66	107.59	112.73	110.33	2.59	2.34%
	9	11.86	12.39	12.43	12.23	0.32	2.60%	26.53	26.46	26.69	26.56	0.12	0.44%	131.69	136.26	135.40	134.45	2.43	1.81%
	10	14.89	14.82	15.16	14.96	0.18	1.20%	27.26	27.19	28.33	27.59	0.64	2.32%	160.82	164.75	162.69	162.75	1.97	1.21%
	11	10.75	10.68	10.72	10.72	0.04	0.33%	23.09	23.32	23.16	23.19	0.12	0.51%	113.46	113.39	116.83	114.56	1.97	1.72%
	12	6.39	6.39	6.46	6.41	0.04	0.63%	18.46	17.89	18.53	18.29	0.35	1.92%	94.09	94.62	96.16	94.96	1.08	1.13%

Table S2. Single-Factor Optimization Results of the Self-Developed High-Salt Lysis Method (Purity：$OD_{260}/_{280}$)

		C.albicans									C.neoformans					
	（CFU/ml）	10^3			10^5			10^7			10^3			10^5		
Concentration of Guanidine Hydrochloride（mol/L）	1	1.94	1.86	1.83	1.85	1.89	1.86	1.81	1.84	1.87	1.82	1.85	1.80	1.82	1.92	1.83
	2	1.88	1.89	1.97	1.81	1.87	1.90	1.88	1.87	1.95	1.87	1.84	1.86	1.88	1.85	1.86
	3	1.92	1.91	1.94	1.92	1.91	1.94	1.92	1.91	1.90	1.91	1.93	1.88	1.92	1.89	1.90
	4	1.77	1.68	1.85	1.87	1.96	1.89	1.87	1.87	1.82	1.86	1.84	1.97	1.87	1.85	1.86
	5	1.73	1.76	1.65	1.83	1.82	1.95	1.83	1.82	1.81	1.82	1.79	1.83	1.83	1.79	1.81
Volume fraction of isopropanol（%）	30	1.84	1.83	1.95	1.84	1.87	1.84	1.84	1.83	1.93	1.85	1.81	1.89	1.85	1.81	1.93
	40	1.89	1.81	1.87	1.89	1.83	1.91	1.89	1.86	1.96	1.89	1.94	1.88	1.79	1.86	1.87
	50	1.93	1.92	1.92	1.93	1.92	1.95	1.83	1.92	1.91	1.94	1.90	1.82	1.94	1.91	1.82
	60	1.86	1.89	1.88	1.86	1.85	1.95	1.86	1.81	1.88	1.86	1.97	1.84	1.86	1.88	1.84
	70	1.82	1.81	1.82	1.82	1.87	1.81	1.86	1.81	1.83	1.82	1.79	1.88	1.82	1.79	1.88
Volume fraction of Triton X-100（%）	0	1.86	1.84	1.94	1.84	1.80	1.83	1.81	1.85	1.83	1.81	1.87	1.80	1.81	1.78	1.85
	2	1.87	1.82	1.85	1.84	1.81	1.89	1.82	1.86	1.94	1.82	1.85	1.89	1.87	1.81	1.85
	4	1.95	1.92	1.87	1.95	1.83	1.97	1.97	1.83	1.97	1.95	1.82	1.94	1.96	1.93	1.85
	6	1.81	1.87	1.93	1.88	1.81	1.92	1.88	1.94	1.90	1.88	1.85	1.86	1.88	1.83	1.86
	8	1.83	1.88	1.83	1.85	1.82	1.85	1.83	1.81	1.81	1.88	1.84	1.89	1.83	1.84	1.81
	10	1.83	1.79	1.82	1.80	1.80	1.82	1.80	1.86	1.82	1.80	1.84	1.77	1.80	1.77	1.82
pH	6	1.80	1.86	1.83	1.88	1.79	1.84	1.80	1.79	1.82	1.80	1.81	1.87	1.80	1.87	1.78
	7	1.85	1.81	1.92	1.93	1.84	1.87	1.95	1.94	1.87	1.85	1.96	1.84	1.85	1.82	1.86
	8	1.89	1.84	1.91	1.89	1.88	1.85	1.83	1.79	1.91	1.89	1.97	1.82	1.90	1.97	1.88
	9	1.87	1.83	1.94	1.92	1.81	1.84	1.92	1.92	1.84	1.92	1.90	1.91	1.93	1.90	1.81
	10	1.96	1.89	1.82	1.84	1.95	1.88	1.96	1.84	1.91	1.97	1.85	1.83	1.83	1.85	1.96
	11	1.91	1.96	1.83	1.91	1.81	1.83	1.94	1.95	1.82	1.91	1.89	1.89	1.91	1.85	1.89
	12	1.86	1.85	1.92	1.86	1.93	1.88	1.86	1.83	1.87	1.86	1.82	1.87	1.86	1.82	1.85

Table S2. Single-Factor Optimization Results of the Self-Developed High-Salt Lysis Method (Purity：$OD_{260}/_{280}$)

	C.neoformans			A.fumigatus								
（CFU/ml）	10^7			10^2			10^4			10^6		
1	1.84	1.82	1.86	1.82	1.81	1.84	1.81	1.83	1.88	1.84	1.83	1.87

Concentration of Guanidine Hydrochloride（mol/L）	2	1.86	1.84	1.88	1.85	1.94	1.82	1.87	1.80	1.83	1.88	1.93	1.89
	3	1.90	1.88	1.92	1.94	1.84	1.95	1.95	1.94	1.83	1.96	1.95	1.83
	4	1.86	1.85	1.87	1.90	1.80	1.97	1.91	1.84	1.97	1.80	1.81	1.92
	5	1.81	1.87	1.93	1.85	1.84	1.83	1.85	1.88	1.83	1.85	1.84	1.93
Volume fraction of isopropanol（%）	30	1.83	1.82	1.86	1.81	1.90	1.82	1.83	1.94	1.84	1.83	1.86	1.84
	40	1.82	1.89	1.88	1.86	1.83	1.87	1.89	1.88	1.89	1.84	1.82	1.90
	50	1.92	1.83	1.93	1.85	1.95	1.84	1.96	1.83	1.82	1.97	1.84	1.86
	60	1.85	1.94	1.86	1.88	1.97	1.86	1.88	1.85	1.89	1.88	1.83	1.89
	70	1.81	1.86	1.82	1.83	1.88	1.84	1.84	1.83	1.86	1.84	1.83	1.82
Volume fraction of Triton X-100（%）	0	1.80	1.85	1.81	1.81	1.81	1.79	1.92	1.81	1.84	1.87	1.81	1.83
	2	1.86	1.85	1.92	1.87	1.84	1.88	1.88	1.85	1.89	1.88	1.87	1.84
	4	1.94	1.83	1.89	1.95	1.98	1.96	1.97	1.94	1.78	1.98	1.84	1.82
	6	1.84	1.93	1.87	1.87	1.86	1.88	1.90	1.83	1.91	1.83	1.85	1.91
	8	1.81	1.88	1.82	1.82	1.81	1.83	1.85	1.82	1.86	1.85	1.84	1.83
	10	1.79	1.98	1.85	1.82	1.80	1.97	1.95	1.80	1.86	1.78	1.98	1.85
pH	6	1.79	1.83	1.86	1.79	1.78	1.84	1.84	1.81	1.83	1.82	1.81	1.83
	7	1.84	1.83	1.85	1.84	1.83	1.80	1.87	1.88	1.88	1.87	1.79	1.88
	8	1.88	1.83	1.81	1.89	1.88	1.93	1.98	1.91	1.91	1.92	1.96	1.93
	9	1.91	1.84	1.83	1.92	1.91	1.93	1.94	1.96	1.95	1.95	1.94	1.91
	10	1.86	1.95	1.87	1.97	1.92	1.98	1.96	1.95	1.97	1.97	1.96	1.98
	11	1.90	1.88	1.92	1.91	1.95	1.92	1.78	1.90	1.83	1.91	1.95	1.92
	12	1.85	1.89	1.86	1.85	1.84	1.81	1.85	1.82	1.86	1.85	1.84	1.86

Table S3. ANOVA Results of the Effects of Different Optimization Factors on Fungal Nucleic Acid Extraction Concentration(medium and high concentration)

Fungal species	Optimization factor	Medium concentration				High concentration			
		**Number of levels**	**F-value**	**P-value**	**optimum level**	**Number of levels**	**F-value**	**P-value**	**optimum level**
C. albicans	Concentration of Guanidine Hydrochloride (mol/L)	5	247.7	<0.0001	3	5	301.3	<0.0001	3
	Volume fraction of isopropanol (%)	5	745.1	<0.0001	50	5	623.0	<0.0001	50
	Volume fraction of Triton X-100 (%)	6	1016	<0.0001	4	6	2519	<0.0001	4
	System pH	7	1527	<0.0001	10	7	2161	<0.0001	10
C. neoformans	Concentration of Guanidine Hydrochloride (mol/L)	5	164.7	<0.0001	3L	5	520.5	<0.0001	3
	Volume fraction of isopropanol (%)	5	386.4	<0.0001	50	5	379.1	<0.0001	50
	Volume fraction of Triton X-100 (%)	6	734.3	<0.0001	4	6	280.3	<0.0001	4
	System pH	7	479.6	<0.0001	10	7	3174	<0.0001	10

A. fumigatus	Concentration of Guanidine Hydrochloride （mol/L）	5	3034	<0.0001	3	5	886.4	<0.0001	3
	Volume fraction of isopropanol （%）	5	706.8	<0.0001	50	5	683.1	<0.0001	50
	Volume fraction of Triton X-100 （%）	6	7847	<0.0001	4	6	324.3	<0.0001	4
	System pH	7	570.6	<0.0001	10	7	609.1	<0.0001	10

Table S4. Extraction Results of Two Different Fungal Nucleic Acid Extraction Methods (Concentration)

	Methods	CFU/ml	Concentration (ng/μL)			Mean (ng/μL)	SD	CV
C.albicans	High-salt	10^3	9.58	9.17	9.43	9.39	0.21	2.21%
		10^5	20.35	20.30	20.31	20.32	0.03	0.13%
		10^7	222.47	217.46	220.62	220.18	2.53	1.15%
	Kit-based	10^3	10.30	10.33	10.33	10.32	0.02	0.17%
		10^5	18.80	18.75	18.73	18.76	0.04	0.19%
		10^7	204.26	201.78	202.52	202.85	1.27	0.63%
C.neoformans	High-salt	10^3	6.52	6.41	6.59	6.51	0.09	1.39%
		10^5	22.05	21.22	21.31	21.53	0.46	2.12%
		10^7	214.58	208.36	210.32	211.09	3.18	1.51%
	Kit-based	10^3	5.64	5.62	5.63	5.63	0.01	0.18%
		10^5	19.75	19.70	19.77	19.74	0.04	0.18%
		10^7	213.66	208.37	210.43	210.82	2.67	1.26%
A.fumigatus	High-salt	10^2	15.34	15.42	15.53	15.43	0.10	0.62%
		10^4	28.45	28.38	28.40	28.41	0.04	0.13%
		10^6	161.47	160.42	160.95	160.95	0.53	0.33%
	Kit-based	10^2	13.70	13.60	13.62	13.64	0.05	0.39%
		10^4	25.55	25.50	25.51	25.52	0.03	0.10%
		10^6	154.84	152.66	153.96	153.82	1.10	0.71%

Table S5. Extraction results of two different fungal nucleic acid extraction methods(purity)

	Methods	CFU/ml	Concentration		
C.albicans	High-salt	10^3	1.81	1.89	1.82
		10^5	1.92	1.92	1.99
		10^7	1.85	1.81	1.85
	Kit-based	10^3	1.97	1.97	1.94
		10^5	1.88	1.84	1.88
		10^7	1.94	1.93	1.91
C.neoformans	High-salt	10^3	1.83	1.86	1.83
		10^5	1.99	1.98	1.96
		10^7	1.87	1.8	1.87
	Kit-based	10^3	1.91	1.95	1.94
		10^5	1.84	1.83	1.89
		10^7	1.96	1.96	1.97
A.fumigatus	High-salt	10^2	1.89	1.88	1.81

Table S5. Extraction results of two different fungal nucleic acid extraction methods(purity)

**Methods**	**CFU/ml**	**Concentration**		
	10^4	1.93	1.91	1.92
	10^6	1.82	1.85	1.84
	10^2	1.98	1.99	1.93
Kit-based	10^4	1.86	1.82	1.86
	10^6	1.9	1.94	1.98

Table S6. Optimization Results of Multiple qPCR Reaction Conditions

		C.albicans						***C.neoformans***					
			**CT**		**Mean**	**SD**	**CV**		**CT**		**Mean**	**SD**	**CV**
Primer concentration (pmol/system)	2	30.98	31.02	30.81	30.94	0.11	0.36%	24.93	24.95	24.99	24.96	0.03	0.12%
	4	30.35	30.35	30.44	30.38	0.05	0.17%	24.43	24.45	24.49	24.46	0.03	0.12%
	6	29.56	29.52	29.54	29.54	0.02	0.07%	24.44	24.47	24.60	24.50	0.09	0.35%
	8	29.3	29.19	29.23	29.24	0.06	0.19%	24.15	24.16	24.06	24.12	0.06	0.23%
	10	29.82	29.83	29.89	29.85	0.04	0.13%	24.97	24.96	24.96	24.96	0.01	0.02%
Probe concentration (pmol/system)	1	29.03	29.12	29.07	29.07	0.05	0.16%	24.46	24.43	24.42	24.44	0.02	0.09%
	2	28.18	28.27	28.35	28.27	0.09	0.30%	24.05	24.12	24.13	24.10	0.04	0.18%
	3	28.64	28.35	28.73	28.57	0.20	0.69%	24.19	24.32	24.36	24.29	0.09	0.37%
	4	28.97	29.13	29.18	29.09	0.11	0.38%	24.14	24.16	24.22	24.17	0.04	0.17%
	5	29.07	29.15	29.22	29.15	0.08	0.26%	24.35	24.48	24.42	24.42	0.07	0.27%
	6	29.35	29.49	29.5	29.45	0.08	0.28%	24.64	24.65	24.64	24.64	0.01	0.02%
$MgCl_2$ concentration (mmol/system)	0	30.64	30.42	30.72	30.59	0.16	0.51%	25.72	24.90	24.81	25.14	0.50	1.99%
	2	30.41	30.30	30.11	30.27	0.15	0.50%	24.72	24.61	24.62	24.65	0.06	0.25%
	4	29.24	29.17	29.4	29.27	0.12	0.40%	24.21	24.24	24.27	24.24	0.03	0.12%
	6	29.56	29.33	29.49	29.46	0.12	0.40%	23.88	23.86	23.95	23.90	0.05	0.20%
	8	29.69	29.23	29.84	29.59	0.32	1.07%	24.65	23.88	23.99	24.17	0.42	1.72%
Annealing Temperature (℃)	54	30.38	30.25	30.28	30.30	0.07	0.22%	24.40	24.38	24.43	24.40	0.03	0.10%
	56	29.88	29.85	30.01	29.91	0.09	0.28%	23.69	23.68	23.73	23.70	0.03	0.11%
	58	28.61	28.67	28.54	28.61	0.07	0.23%	23.43	23.31	23.44	23.39	0.07	0.31%
	60	28.23	28.70	28.88	28.60	0.34	1.17%	23.95	23.94	23.95	23.95	0.01	0.02%
	62	28.91	28.77	28.99	28.89	0.11	0.39%	24.55	24.58	24.58	24.57	0.02	0.07%

Table S6. Optimization Results of Multiple qPCR Reaction Conditions

		A.fumigatus					
			**CT**		**Mean**	**SD**	**CV**
	2	30.98	31.02	30.81	30.94	0.11	0.36%

Primer concentration（pmol/system）	4	30.35	30.35	30.44	30.38	0.05	0.17%
	6	29.56	29.52	29.54	29.54	0.02	0.07%
	8	29.3	29.19	29.23	29.24	0.06	0.19%
	10	29.82	29.83	29.89	29.85	0.04	0.13%
Probe concentration（pmol/system）	1	29.03	29.12	29.07	29.07	0.05	0.16%
	2	28.18	28.27	28.35	28.27	0.09	0.30%
	3	28.64	28.35	28.73	28.57	0.20	0.69%
	4	28.97	29.13	29.18	29.09	0.11	0.38%
	5	29.07	29.15	29.22	29.15	0.08	0.26%
	6	29.35	29.49	29.5	29.45	0.08	0.28%
MgCl2 concentration（mmol/system）	0	30.64	30.42	30.72	30.59	0.16	0.51%
	2	30.41	30.30	30.11	30.27	0.15	0.50%
	4	29.24	29.17	29.4	29.27	0.12	0.40%
	6	29.56	29.33	29.49	29.46	0.12	0.40%
	8	29.69	29.23	29.84	29.59	0.32	1.07%
Annealing Temperature（℃）	54	30.38	30.25	30.28	30.30	0.07	0.22%
	56	29.88	29.85	30.01	29.91	0.09	0.28%
	58	28.61	28.67	28.54	28.61	0.07	0.23%
	60	28.23	28.70	28.88	28.60	0.34	1.17%
	62	28.91	28.77	28.99	28.89	0.11	0.39%

Table S7. Sensitivity Results of Single qPCR Reaction System

	(CFU/ml)		CT		Mean	SD	CV
C.albicans	10^1	43.8	NoCt	42.84	43.32	0.68	1.57%
	10^2	41.59	41.64	41.43	41.55	0.11	0.26%
	10^3	37.8	37.67	38.23	37.90	0.29	0.77%
	10^4	34.76	35.44	34.63	34.94	0.44	1.24%
	10^5	31.04	31.18	31.16	31.13	0.08	0.24%
	10^6	27.89	28.01	27.86	27.92	0.08	0.28%
	10^7	23.65	23.8	23.75	23.73	0.08	0.32%
	10^8	19.92	19.96	20.09	19.99	0.09	0.44%
C.neoformans	10^1	40.14	40.12	NoCt	40.13	0.01	0.04%
	10^2	37.06	37.21	37.13	37.13	0.08	0.20%
	10^3	33.95	34.21	34.39	34.18	0.22	0.65%
	10^4	31.44	31.24	31.37	31.35	0.10	0.32%
	10^5	28.05	28.76	28.11	28.31	0.39	1.39%
	10^6	25.35	25.39	25.16	25.30	0.12	0.49%
	10^7	20.78	20.82	20.69	20.76	0.07	0.32%
	10^8	17.68	17.58	17.76	17.67	0.09	0.51%
A.fumigatus	10^1	35.34	35.12	34.93	35.13	0.21	0.58%
	10^2	30.67	30.46	30.61	30.58	0.11	0.35%
	10^3	26.95	26.91	26.79	26.88	0.08	0.31%
	10^4	24.44	24.94	24.68	24.69	0.25	1.01%
	10^5	21.05	21.11	25.26	22.47	2.41	10.74%
	10^6	16.35	16.39	16.75	16.50	0.22	1.34%

Table S8. Sensitivity and Reproducibility Results of the Multiplex qPCR Reaction System

		First times within the batch						Second time within the batch					
	（CFU/ml）		CT		Mean	SD	CV		CT		Mean	SD	CV
C.albicans	10^1	42.34	NoCt	41.67	42.01	0.47	1.13%	41.96	NoCt	43.15	42.56	0.84	1.98%
	10^2	40.69	40.13	39.97	40.26	0.38	0.94%	40.45	40.36	40.22	40.34	0.12	0.29%
	10^3	36.32	35.80	34.87	35.66	0.73	2.06%	34.95	35.10	34.85	34.97	0.13	0.36%
	10^4	33.54	33.65	33.73	33.64	0.10	0.28%	32.47	32.50	32.60	32.52	0.07	0.21%
	10^5	29.04	27.86	28.86	28.59	0.64	2.22%	28.90	28.35	28.32	28.52	0.33	1.14%
	10^6	26.69	26.80	26.46	26.65	0.17	0.65%	26.80	26.70	26.65	26.72	0.08	0.29%
	10^7	23.15	23.35	23.35	23.28	0.12	0.50%	23.45	23.30	23.50	23.42	0.10	0.44%
	10^8	18.92	18.68	19.19	18.93	0.26	1.35%	18.85	18.80	19.05	18.90	0.13	0.70%
C.neoformans	10^1	41.32	40.26	NoCt	40.79	0.75	1.84%	42.83	41.72	NoCt	42.28	0.78	1.86%
	10^2	37.89	37.26	37.75	37.63	0.33	0.88%	38.21	37.88	37.53	37.87	0.34	0.90%
	10^3	34.95	34.21	34.53	34.56	0.37	1.07%	35.13	34.46	35.33	34.97	0.46	1.30%
	10^4	32.44	32.44	33.37	32.75	0.54	1.64%	32.17	33.95	32.84	32.99	0.90	2.73%
	10^5	28.54	28.14	28.05	28.24	0.26	0.92%	27.56	27.39	27.81	27.59	0.21	0.77%
	10^6	25.95	25.89	26.62	26.15	0.41	1.55%	26.54	25.33	26.36	26.08	0.65	2.50%
	10^7	22.78	22.82	21.66	22.42	0.66	2.94%	22.23	23.36	22.56	22.72	0.58	2.56%
	10^8	18.48	18.52	17.96	18.32	0.31	1.71%	18.17	19.82	18.78	18.92	0.83	4.41%
A.fumigatus	10^1	34.88	34.27	34.75	34.63	0.32	0.93%	34.73	34.29	34.57	34.53	0.22	0.64%
	10^2	31.67	29.89	31.64	31.07	1.02	3.28%	31.92	31.64	32.79	32.12	0.60	1.87%
	10^3	27.05	27.11	26.79	26.98	0.17	0.63%	27.27	27.26	27.04	27.19	0.13	0.48%
	10^4	23.44	23.94	23.68	23.69	0.25	1.06%	23.59	23.79	23.68	23.69	0.10	0.42%
	10^5	20.26	20.11	20.12	20.16	0.08	0.42%	20.51	19.96	20.17	20.21	0.28	1.37%
	10^6	17.85	17.41	17.75	17.67	0.23	1.31%	17.97	17.66	17.87	17.83	0.16	0.89%

Table S8. Sensitivity and Reproducibility Results of the Multiplex qPCR Reaction System

		Three times within the batch						Between batches					
	（CFU/ml）	CT			Mean	SD	CV	CT			Mean	SD	CV
C.albicans	10^1	41.74	41.33	42.61	41.89	0.65	1.56%	42.01	42.56	41.89	42.15	0.35	0.84%
	10^2	39.18	38.39	40.18	39.25	0.90	2.29%	40.26	40.34	39.25	39.95	0.61	1.53%
	10^3	34.59	35.13	34.54	34.75	0.33	0.94%	35.66	34.97	34.75	35.13	0.48	1.35%
	10^4	32.61	32.84	31.92	32.46	0.48	1.48%	33.64	32.52	32.46	32.87	0.66	2.02%
	10^5	28.95	29.18	28.69	28.94	0.25	0.85%	28.59	28.52	28.94	28.68	0.22	0.78%
	10^6	26.73	26.96	27.68	27.12	0.50	1.83%	26.65	26.72	27.12	26.83	0.26	0.95%
	10^7	23.18	23.68	22.97	23.28	0.36	1.57%	23.28	23.42	23.28	23.33	0.08	0.34%
	10^8	19.18	18.79	18.58	18.85	0.30	1.62%	18.93	18.90	18.85	18.89	0.04	0.21%
C.neoformans	10^1	42.63	41.52	41.97	42.04	0.56	1.33%	40.79	42.28	42.04	41.70	0.80	1.91%
	10^2	37.65	37.53	38.12	37.77	0.31	0.83%	37.63	37.87	37.77	37.76	0.12	0.32%
	10^3	34.67	34.86	34.81	34.78	0.10	0.28%	34.56	34.97	34.78	34.77	0.21	0.59%
	10^4	33.70	32.97	32.63	33.10	0.55	1.65%	32.75	32.99	33.10	32.95	0.18	0.54%
	10^5	27.47	28.26	28.34	28.02	0.48	1.72%	28.24	27.59	28.02	27.95	0.33	1.20%
	10^6	25.38	25.35	26.34	25.69	0.56	2.19%	26.15	26.08	25.69	25.97	0.25	0.96%
	10^7	21.46	22.57	22.58	22.20	0.64	2.90%	22.42	22.72	22.20	22.45	0.26	1.15%
	10^8	19.36	19.45	18.73	19.18	0.39	2.05%	18.32	18.92	19.18	18.81	0.44	2.35%
A.fumigatus	10^1	35.28	34.64	33.95	34.62	0.67	1.92%	34.63	34.53	34.62	34.60	0.06	0.16%
	10^2	32.18	31.58	32.86	32.21	0.64	1.99%	31.07	32.12	32.21	31.80	0.63	1.99%
	10^3	28.36	28.49	27.49	28.11	0.54	1.93%	26.98	27.19	28.11	27.43	0.60	2.19%
	10^4	22.49	23.18	22.62	22.76	0.37	1.61%	23.69	23.69	22.76	23.38	0.53	2.28%
	10^5	20.38	19.94	20.74	20.35	0.40	1.97%	20.16	20.21	20.35	20.24	0.10	0.49%
	10^6	17.46	18.31	17.28	17.68	0.55	3.11%	17.67	17.83	17.68	17.73	0.09	0.51%

Table S9. Sensitivity and Reproducibility Results of the Multiplex qPCR Reaction System

Strain	CFU/mL	Within the batch		Between batches	
		CT（mean±standard deviation）	Coefficient of Variation（CV/%）	CT（mean±standard deviation）	Coefficient of Variation（CV/%）
C. albicans	1×10^1	42.01±0.47	1.13%	42.15±0.35	0.84%
	1×10^2	40.26±0.38	0.94%	39.95±0.61	1.53%
	1×10^3	35.66±0.73	2.06%	35.13±0.48	1.35%
	1×10^4	33.64±0.10	0.28%	32.87±0.66	2.02%
	1×10^5	28.59±0.64	2.22%	28.68±0.22	0.78%
	1×10^6	26.65±0.17	0.65%	26.83±0.26	0.95%
	1×10^7	23.28±0.12	0.50%	23.33±0.08	0.34%
	1×10^8	18.93±0.26	1.35%	18.89±0.04	0.21%
C. neoformans	1×10^1	40.79±0.75	1.84%	41.70±0.80	1.91%
	1×10^2	37.63±0.33	0.88%	37.76±0.12	0.32%
	1×10^3	34.56±0.37	1.07%	34.77±0.21	0.59%
	1×10^4	32.75±0.54	1.64%	32.95±0.18	0.54%
	1×10^5	28.24±0.26	0.92%	27.95±0.33	1.20%
	1×10^6	26.15±0.41	1.55%	25.97±0.25	0.96%
	1×10^7	22.42±0.66	2.94%	22.45±0.26	1.15%
	1×10^8	18.32±0.31	1.71%	18.81±0.44	2.35%
A. fumigatus	1×10^1	42.01±0.47	1.13%	42.15±0.35	0.84%
	1×10^2	40.26±0.38	0.94%	39.95±0.61	1.53%
	1×10^3	35.66±0.73	2.06%	35.13±0.48	1.35%
	1×10^4	33.64±0.10	0.28%	32.87±0.66	2.02%
	1×10^5	28.59±0.64	2.22%	28.68±0.22	0.78%
	1×10^6	26.65±0.17	0.65%	26.83±0.26	0.95%
